# Store-independent modulation of Ca^2+^ entry through Orai by Septin 7

**DOI:** 10.1038/ncomms11751

**Published:** 2016-05-26

**Authors:** Bipan Kumar Deb, Trayambak Pathak, Gaiti Hasan

**Affiliations:** 1 National Centre for Biological Sciences, Tata Institute of Fundamental Research, Bellary Road, Bangalore 560065, India; 2 Manipal University, Manipal, Karnataka 576104, India

## Abstract

Orai channels are required for store-operated Ca^2+^ entry (SOCE) in multiple cell types. Septins are a class of GTP-binding proteins that function as diffusion barriers in cells. Here we show that Septin 7 acts as a ‘molecular brake’ on activation of Orai channels in *Drosophila* neurons. Lowering Septin 7 levels results in dOrai-mediated Ca^2+^ entry and higher cytosolic Ca^2+^ in resting neurons. This Ca^2+^ entry is independent of depletion of endoplasmic reticulum Ca^2+^ stores and Ca^2+^ release through the inositol-1,4,5-trisphosphate receptor. Importantly, store-independent Ca^2+^ entry through Orai compensates for reduced SOCE in the *Drosophila* flight circuit. Moreover, overexpression of Septin 7 reduces both SOCE and flight duration, supporting its role as a negative regulator of Orai channel function *in vivo*. Septin 7 levels in neurons can, therefore, alter neural circuit function by modulating Orai function and Ca^2+^ homeostasis.

## Abstract (web summary)

Orai channels are well known to mediate store-operated calcium entry. Here authors show that in neurons of the *Drosophila* flight circuit, Septin 7 acts as a negative regulator of Orai channels, surprisingly, by modulating store-independent calcium entry through Orai.

## Graphical abstract

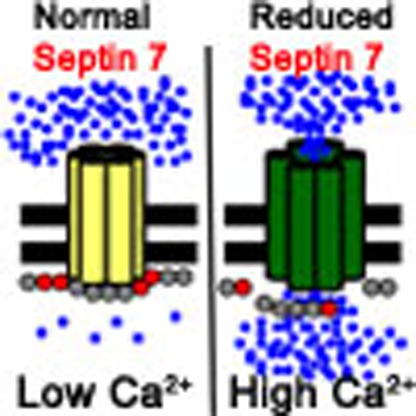

Physiological signals such as neurotransmitters, hormones and growth factors frequently regulate cellular function by the release of intracellular calcium from endoplasmic reticulum (ER) stores, followed by store-operated calcium entry (SOCE)^1^. This calcium signalling mechanism can be initiated by surface receptors that are coupled with phospholipase C, which generates inositol-1,4,5-trisphosphate (IP_3_) from plasma membrane (PM)-localized phosphatidyl inositol-4,5 bisphosphate or PIP_2_ (ref. 2). IP_3_ binds to and opens a ligand-gated intracellular Ca^2+^ channel, the IP_3_ receptor (IP_3_R) present on the membranes of ER stores, thus leading to the release of intracellular Ca^2+^^2^. The consequent drop in intracellular store Ca^2+^ is sensed by ER membrane-localized stromal interaction molecule (STIM) proteins, which subsequently oligomerize and translocate to the ER closely apposed to the PM^3^,^4^,^5^,^6^, where they bind to and open the Orai channel, leading to SOCE^7^,^8^,^9^,^10^,^11^,^12^.

Whereas STIM and Orai constitute integral components of SOCE, additional proteins regulate the strength and duration of SOCE signals^1^,^13^. Molecular screens for SOCE regulators have identified both positive^13^,^14^,^15^,^16^,^17^,^18^ and negative^19^,^20^ regulators of SOCE, some of which like SOCE associated regulatory factor (SARAF)^19^ are specific for vertebrates. Genetic studies in *Drosophila*^21^,^22^,^23^ have shown that IP_3_R mutants as well as flies with pan-neuronal knockdown of IP_3_R, dSTIM or dOrai exhibit reduced flight bouts^21^,^22^. Importantly, flight deficits of IP_3_R mutants can be rescued by overexpression of dSTIM and dOrai in neurons, indicating that IP_3_R-mediated Ca^2+^ release is coupled with SOCE through dSTIM/dOrai in neurons of the *Drosophila* flight circuit. *Drosophila* mutants for the IP_3_R (the gene is referred to as *itpr*) thus offer a genetic background for identifying additional regulators of SOCE via dSTIM/dOrai whose effect on IP_3_-mediated Ca^2+^ release and SOCE can be tested in cultured primary neurons derived from the central nervous system. Moreover, their physiological relevance can be validated *in vivo* by quantification of IP_3_R mutant flight deficits^24^ on introduction of mutants or overexpression constructs of the putative interacting partners.

A recent screen in HeLa cells identified mammalian septins, SEPT2, SEPT4 and SEPT5, as positive regulators of SOCE^25^. Septins are a class of evolutionarily conserved filament forming GTPases that primarily function as molecular adaptors or diffusion barriers in cells^26^. Five septin-encoding genes are reported in *Drosophila*^27^,^28^,^29^ among which the ‘*pnut*’ gene^27^ encodes the *Drosophila* homologue of mammalian Septin 7 (ref. 29), henceforth referred to as dSEPT7 (the gene is referred to as *dSEPT7*). Both in *Drosophila* and mammals, the SEPT7 family is represented by a single gene^26^,^29^ (Table 1). To investigate whether regulation of SOCE by septins is conserved in *Drosophila* neurons, a deficiency allele of the *dSEPT7* gene, *pnut*^*xp*^ (ref. 27), was tested for genetic interaction with *itpr* mutants. Systemic and cellular studies demonstrate a novel role for dSEPT7 in regulation of dOrai-mediated Ca^2+^ entry in neurons.

## Results

### dSEPT7 reduction can compensate for reduced IP_3_R function

**Figure 1:**
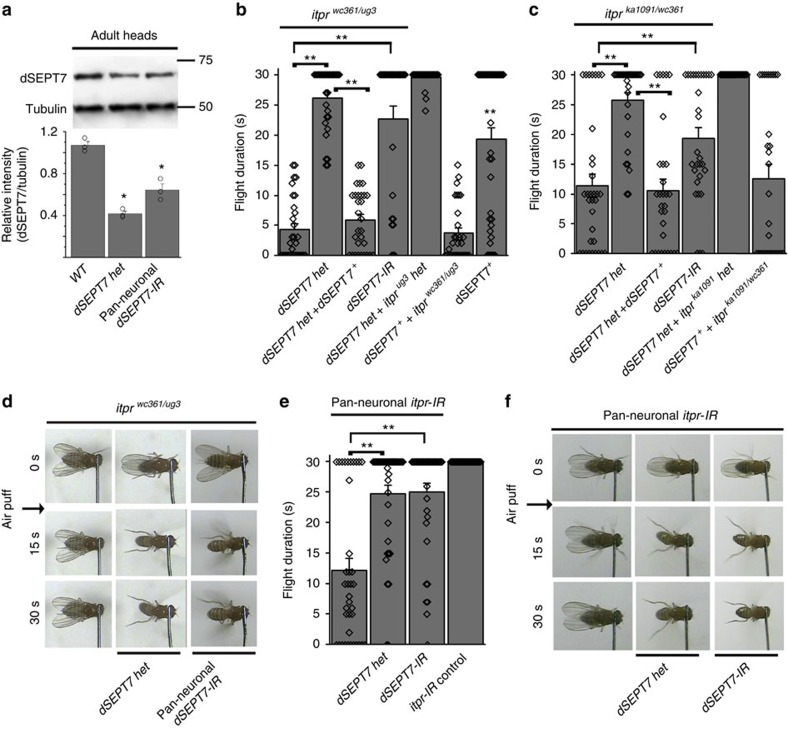
Flight deficits in IP_3_R mutants and knockdown flies can be modulated by dSEPT7 expression. (**a**) A representative western blot and quantification of three such blots measuring dSEPT7 protein levels in extracts from heads of adult flies of the indicated genotypes. dSEPT7 levels are significantly reduced in ‘*dSEPT7 het*’ flies and in flies with pan-neuronal knockdown of *dSEPT7* when compared with the wild type (Canton-S); *P<0.05, Student’s *t*-test, *N*=3. (**b**,**c**) Flight duration in two *itpr* mutant strains tested, *itpr*^*wc361/ug3*^ (**b**) and *itpr*^*ka1091/wc361*^ (**c**), improved significantly on reducing dSEPT7 with either a single copy of the deficiency allele, ‘*dSEPT7 het*’, or pan-neuronal knockdown of *dSEPT7* (*dSEPT7-IR*) in neurons. Overexpression of dSEPT7 (*dSEPT7*^*+*^) in neurons restored flight deficits in *itpr*^*wc361/ug3*^ or *itpr*^*ka1091/wc361*^ flies with reduced dSEPT7 (*dSEPT7 het*+*itpr*^*wc361/ug3*^ or *dSEPT7 het*+*itpr*^*ka1091/wc361*^). The pan-neuronal *elav*^*C155*^*GAL4* driver was used for expressing *dSEPT7-IR* or *dSEPT7*^*+*^ in all neurons of the fly. Flight times of 30 or more flies, monitored for 30 s are shown as mean±s.e.m. The diamonds within each bar represent the flight duration of a single fly. **P<0.01, Mann–Whitney *U*-test with Bonferroni correction. Brackets indicate the genotypes being compared. Flight times in *dSEPT7*^*+*^ animals are compared with wild-type animals (shown in Supplementary Fig. 1b). (**d**) Snapshots from video recordings of air-puff-induced flight of the indicated genotypes at 0 s (start), 15 and 30 s after delivery of the air-puff. (**e**) Reduced flight times observed by pan-neuronal knockdown of *itpr* with an *RNAi* (*itpr-IR*) were rescued by reduction of dSEPT7 with either a single copy of the deficiency allele, ‘*dSEPT7 het*’ or pan-neuronal knockdown of *dSEPT7. itpr-IR control* refers to animals with the *itpr-IR* transgene, but no *GAL4* driver, resulting in no expression of the RNAi and no reduction in IP_3_R levels (Supplementary Fig. 1e). **P<0.01, Mann–Whitney *U*-test with Bonferroni correction. (**f**) Snapshots from video recordings of air-puff-induced flight of the indicated genotypes at 0 s (start), 15 and 30 s after delivery of the air-puff.

Animals homozygous for the *dSEPT7* deficiency allele (*pnut^xp/xp^*, here after referred to as ‘*dSEPT7* null’) are lethal during development and fail to survive till adulthood^27^. In animals heterozygous for this deficiency allele (*pnut*^*xp/+*^, hereafter referred to as *‘dSEPT7 het*’), dSEPT7 protein levels are reduced to ∼50% of wild-type (WT) levels in adult heads and the larval central nervous system (CNS) (Fig. 1a; Supplementary Fig. 1c). The role of dSEPT7 in intracellular Ca^2+^ signalling was investigated by introducing a single copy of the deficiency allele for dSEPT7 (ref. 27; *dSEPT7 het*) in two viable IP_3_R mutant combinations, *itpr*^*wc361/ug3*^ and *itpr*^*ka1091/wc361*^ (refs 24, 30). The *wc361* allele has a deletion of 15 residues at the C terminus due to the mutation of an arginine residue at position 2,814 to a stop codon. The *ug3* allele converts a serine to a phenylalanine at position 224 in the ligand-binding domain of the *Drosophila* IP_3_R, whereas in the *ka1091* mutant allele, a glycine residue at position 1,891 in the modulatory domain of the protein is mutated to a serine^30^. As with other viable combinations of the IP_3_R, the heteroallelic combinations tested here exhibit defective wing postures and significantly reduced flight durations (Fig. 1b–d; Supplementary Fig. 1a), but appear normal as heterozygotes^24^ (Supplementary Fig. 1b). Interestingly, introduction of a single copy of the *dSEPT7* deletion allele rescued flight (Fig. 1b–d) and wing-posture defects (Supplementary Fig. 1a) to a significant extent in both *itpr* mutants. Control genotypes consisting of *dSEPT7 het* in combination with either WT or heterozygotes of *itpr* gene mutants exhibit normal flight durations (Fig. 1b; Supplementary Fig. 1b). The focus of this genetic interaction is the nervous system because targeted reduction of dSEPT7 in neurons with an RNA interference (RNAi; *dSEPT7-IR*) also rescued flight deficits of the tested *itpr* mutants (Fig. 1b–d). Moreover, flight deficits of animals where IP_3_R was reduced in the CNS by pan-neuronal knockdown with an RNAi against *itpr* (*itpr-IR*)^22^,^31^ could be rescued by knockdown of dSEPT7 or partial genetic depletion of *dSEPT7* (Fig. 1e,f; Supplementary Fig. 1a). *dSEPT7-IR* significantly reduced dSEPT7 protein in lysates from both adult fly heads and larval CNS (Fig. 1a; Supplementary Fig. 1c). Together, these data suggest that partial loss of dSEPT7 compensates for the reduced IP_3_R function in neurons of the *Drosophila* flight circuit. Conversely, restoring dSEPT7 levels by pan-neuronal overexpression of a *dSEPT7*^*+*^ transgene in *itpr* mutant animals heterozygous for *dSEPT7* (*dSEPT7 het*+*itpr*^*mutant*^) reverted the rescue obtained on reducing dSEPT7 and resulted in flies with flight and wing-posture defects similar to *itpr* mutants (Fig. 1b–d; Supplementary Fig. 1a). dSEPT7 overexpression in neurons of WT flies also resulted in significant flight deficits, suggesting a negative regulatory function of dSEPT7 in flight circuit neurons (Fig. 1b; Supplementary Fig. 1d). The genetic interactions observed between dSEPT7 and IP_3_R are not due to the changes in expression of the interacting genes, because *itpr* knockdown did not alter dSEPT7 levels in adult fly heads (Supplementary Fig. 1d) and reducing dSEPT7 levels in flies with pan-neuronal knockdown of IP_3_R did not alter the extent of IP_3_R knockdown (Supplementary Fig. 1e). The cellular basis of the genetic interaction between dSEPT7 and IP_3_R was investigated next.

### Reduced dSEPT7 increases the uptake of extracellular Ca^2+^

**Figure 2:**
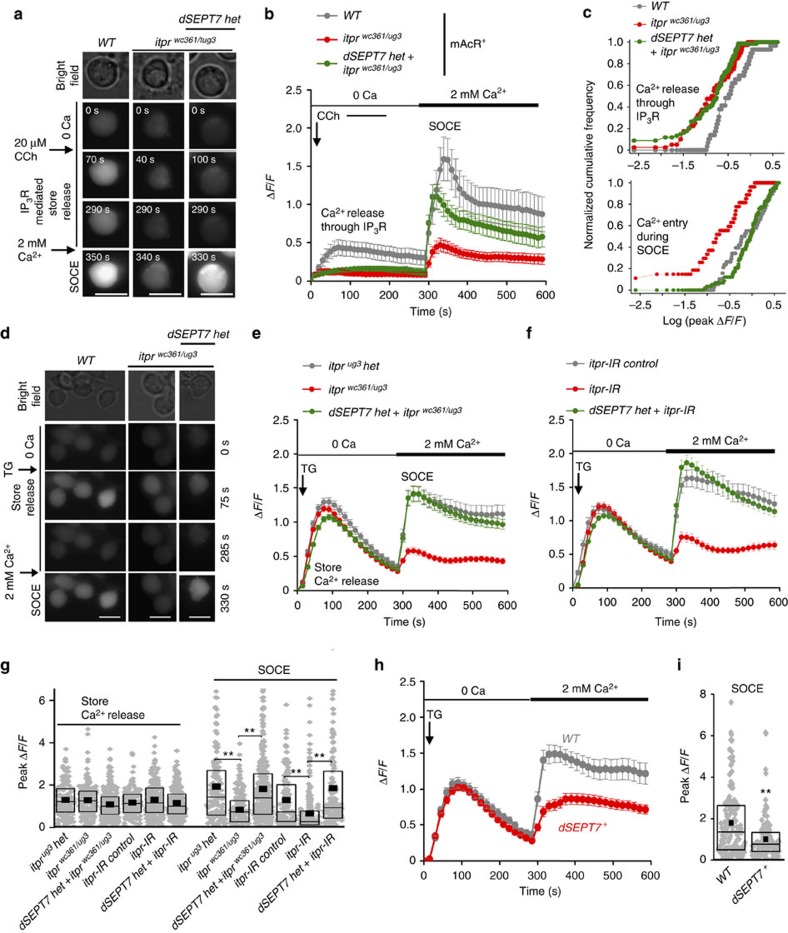
dSEPT7 reduction improves uptake of extracellular Ca^2+^ in neurons with reduced IP_3_R function. (**a**) Representative images of primary *Drosophila* larval neurons of the indicated genotypes. Images were obtained before and after carbachol (CCh, 20 μM)-induced ER store Ca^2+^ release and entry of extracellular Ca^2+^ by SOCE at the indicated time points. Scale bar, 5 μm. (**b**) SOCE in *itpr*^*wc361/ug3*^ neurons with and without dSEPT7 reduction. Traces represent the mean (±s.e.m.) Δ*F*/*F* values obtained at each time point during CCh-induced ER store Ca^2+^ release and SOCE for the indicated genotypes (*N*≥70 cells). (**c**) Kolmogorov–Smirnov (K–S) plots comparing the distribution of peak Δ*F*/*F* values obtained during IP_3_R-mediated Ca^2+^ release (top) and Ca^2+^ entry during SOCE (bottom) in the indicated genotypes. *P*<0.0001, K–S test for *itpr*^*wc361/ug3*^ and ‘*dSEPT7 het*+*itpr*^*wc361/ug3*^’ as compared with WT, (top) and *itpr*^*wc361/ug3*^compared with WT and ‘*dSEPT7 het*+*itpr*^*wc361/ug3*^’ (bottom). (**d**) Representative images of primary *Drosophila* larval neurons of the indicated genotypes with changes in cytosolic Ca^2+^ levels obtained after ER store depletion by thapsigargin (TG, 10 μM) and entry of extracellular Ca^2+^ during SOCE. Scale bar, 5 μm. (**e**,**f**) Reduced SOCE observed in *itpr*^*wc361/ug3*^ neurons (**e**) and neurons with *itpr* knockdown (*itpr-IR*) (**f**) was restored to control levels on reduction of dSEPT7. *itpr*^*ug3*^ *het* (or *itpr*^*ug3/+*^) refers to animals heterozygous for the *ug3* mutation in the *itpr* gene and is a genotypic control for the *itpr*^*wc361/ug3*^ mutant. Pan-neuronal *elav*^*C155*^*GAL4* was used to drive expression of the *itprRNAi (itpr-IR)*. *itpr-IR control* refers to neurons with the RNAi construct, but no GAL4 driver, resulting in the absence of RNAi expression. Traces represent the mean (±s.e.m.) Δ*F*/*F* values obtained at each time point after TG-induced ER store Ca^2+^ depletion and SOCE for the indicated genotypes (*N*≥150 cells). (**g**) Quantification of peak Δ*F*/*F* values obtained by ER store Ca^2+^ depletion (0–285 s) and SOCE (300–600 s) for the indicated genotypes; **P<0.001, Mann–Whitney *U*-test with Bonferroni correction. (**h**) Reduced SOCE in primary neurons with overexpression of *dSEPT7*^*+*^; *N*≥150 cells. *dSEPT7*^*+*^ was expressed using the pan-neuronal *elav*^*C155*^ *GAL4*. (**i**) Quantification of peak Δ*F*/*F* values obtained during SOCE in the indicated genotypes; **P<0.01, Mann–Whitney *U*-test. Diamonds represent peak Δ*F*/*F* values of individual cells.

Ca^2+^ release through the IP_3_R was measured by agonist stimulation of the muscarinic acetylcholine receptor with carbachol (CCh) in primary cultured neurons (Fig. 2a–c)^21^. An initial peak of intracellular Ca^2+^ release through the IP_3_R was observed on CCh stimulation in WT neurons. This peak was significantly reduced in *itpr*^*wc361/ug3*^ mutant neurons (Fig. 2a–c). Reduction of dSEPT7 did not affect Ca^2+^ release through the IP_3_R in WT or *itpr*^*wc361/ug3*^ neurons (Fig. 2a–c; Supplementary Fig. 2a,b). SOCE was observed on Ca^2+^ add-back in the extracellular medium as a second Ca^2+^ peak in WT neurons (Fig. 2a–c; Supplementary Fig. 2b). SOCE induced by IP_3_R-mediated ER store depletion was significantly lower in *itpr* mutant neurons, when compared with WT (Fig. 2a–c; Supplementary Fig. 2b). Interestingly, dSEPT7 reduction resulted in significant uptake of extracellular Ca^2+^ in *itpr* mutant neurons, although the Ca^2+^ release through the mutant IP_3_R remained low and unaltered (Fig. 2b,c). Thus, reducing levels of dSEPT7 restores extracellular Ca^2+^ entry in *itpr* mutant neurons despite attenuated depletion of store Ca^2+^ (Fig. 2a–c; Supplementary Fig. 2b).

Neurons cultured from an *itpr* mutant, *itpr*^*ka1091/ug3*^, exhibit reduced SOCE after passive depletion of ER Ca^2+^ stores by thapsigargin (TG), an inhibitor of the sarco-endoplasmic reticulum Ca^2+^ATPase pump^21^,^23^.This finding contrasts with previous reports from non-excitable DT-40 cells^32^. TG-induced SOCE was reduced in neurons cultured from *itpr*^*wc361/ug3*^ larvae and in neurons with knockdown of the IP_3_R (Fig. 2d–g). dSEPT7 reduction, in either *itpr* mutant or knockdown neurons, significantly improved extracellular Ca^2+^ entry after TG-mediated store depletion, without affecting passive ER Ca^2+^ release (Fig. 2e–g). The cellular specificity of this rescue was tested next. dSEPT7 knockdown with *dSEPT7* RNAi significantly improved extracellular Ca^2+^ entry in *itpr*^*wc361/ug3*^ neurons (Supplementary Fig. 2c,d). Second, overexpression of dSEPT7 reverted rescue of Ca^2+^ entry in *itpr*^*wc361/ug3*^ neurons with reduced dSEPT7 back to the reduced levels observed in *itpr*^*wc361/ug3*^ neurons (Supplementary Fig. 2c,d). Initial rate of Ca^2+^ entry on Ca^2+^ add-back was significantly reduced in both *itpr* mutant and knockdown neurons, and was rescued significantly in neurons with dSEPT7 reduction (Supplementary Fig. 2e). This increase in the rate of Ca^2+^ entry is possibly a consequence of faster dOrai channel activation in dSEPT7 reduced neurons when compared with IP_3_R mutant or knockdown neurons. Together, these data demonstrate that dSEPT7 reduction facilitates extracellular Ca^2+^ entry after either passive or IP_3_R-mediated ER store Ca^2+^ release. The effect of increasing dSEPT7 levels on neuronal SOCE was investigated next.

### Overexpression of dSEPT7 reduces SOCE in neurons

SOCE obtained after TG-mediated passive store depletion in *dSEPT7 het*, *dSEPT7 null* or *dSEPT7-IR* neurons was not significantly different from that of WT neurons (Supplementary Fig. 2f,g). However, overexpression of dSEPT7 resulted in significant reduction of SOCE (Fig. 2h,i). These data, together with the earlier observation (Fig. 2b,d,e), suggest that dSEPT7 could function as a negative regulator of SOCE in neurons. We have earlier demonstrated that upregulation of the SOCE components dSTIM and dOrai in neurons improve SOCE and restore flight in a flightless *itpr* mutant^22^. In agreement with this, expression of *dSTIM*^*+*^ significantly improved SOCE after passive store depletion in neurons with *itpr* knockdown (Supplementary Fig. 2h,i). It is possible that dSEPT7 affects Ca^2+^ entry by regulating levels of SOCE components. dSTIM and dOrai levels were, however, unchanged on either reduction or overexpression of dSEPT7 (Supplementary Fig. 2j). These data support a direct effect of dSEPT7 on the function of either one or both of these SOCE proteins.

**Figure 3:**
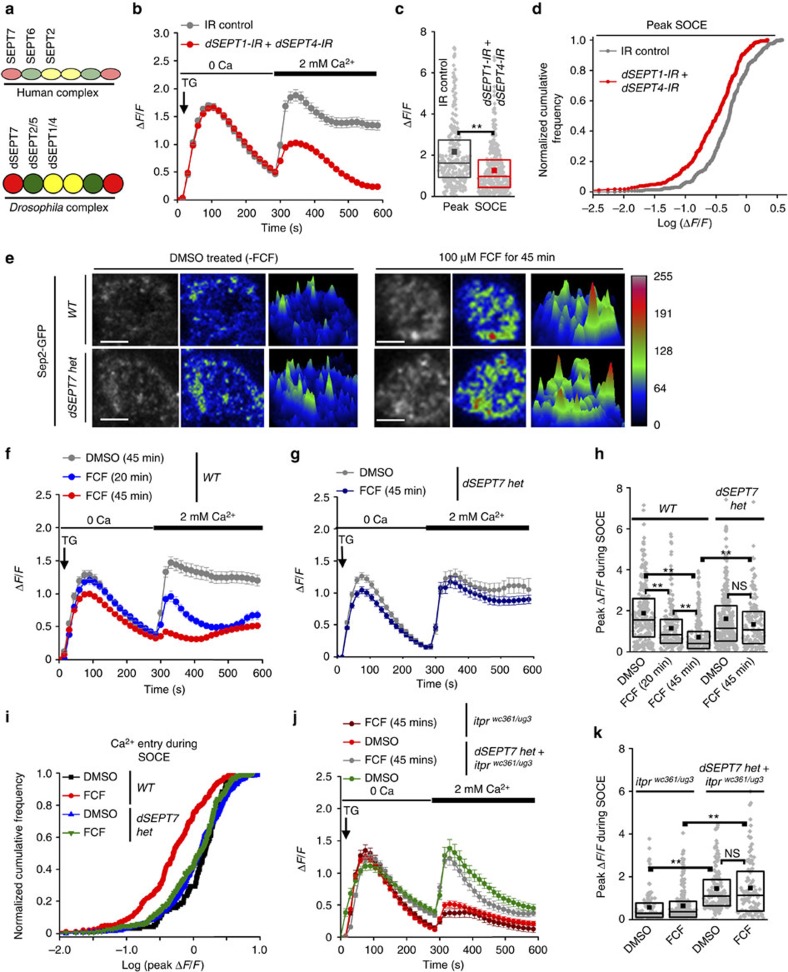
Reducing dSEPT7 restores Ca^2+^ entry in neurons with pharmacological inhibition of septin dynamics. (**a**) Positions occupied by septins of different subgroups (Table 1) in a septin complex formed by human septin subunits and their *Drosophila* homologues. (**b**) SOCE after TG-induced depletion of ER stores in neurons with knockdown of *dSEPT1* and *dSEPT4*. *IR control* refers to neurons carrying RNAi constructs for both genes without the GAL4 driver. *N*>300 cells for each genotype. (**c**) Quantification of peak Δ*F*/*F* values obtained during SOCE. (**d**) K–S plots analysing peak Δ*F*/*F* values during SOCE in the indicated genotypes. The distribution for neurons with *dSEPT1* and *dSEPT4* knockdown is significantly shifted to the left compared with the IR control indicating a greater proportion of cells with lower Δ*F*/*F* values. *P*<0.001, K–S test. (**e**) Sep2-GFP localization in WT and neurons with reduced dSEPT7 treated with either DMSO (control) or 100 μM FCF for 45 min. Original grey scale images have been pseudo-coloured for visualizing the intensity of Sep2-GFP distribution where warmer colours represent higher intensities. A surface plot constructed with the pseudo-coloured image is shown for better resolution. The calibration bar represents the grey scale intensities corresponding to each colour. Scale bar, 3 μm. (**f**,**g**) Changes in Δ*F*/*F* (corresponding to changes in cytosolic [Ca^2+^]) during TG (10 μM)-induced passive ER Ca^2+^ release and SOCE in neurons of the indicated genotypes treated with 100 μM FCF for 20 or 45 min or DMSO for 45 min. *N*>100 cells. (**h**) Box plots quantifying peak Δ*F*/*F* values during SOCE in neurons of the indicated genotypes with or without FCF treatment; **P<0.001, Mann–Whitney *U*-test with Bonferroni correction. (**i**) K–S plots analysing peak Δ*F*/*F* values during SOCE in the indicated genotypes and treatment conditions. *P*<0.0001, K–S test. (**j**) Changes in cytosolic [Ca^2+^] indicated as Δ*F*/*F* during TG (10 μM)-induced passive ER Ca^2+^ release and SOCE in neurons of the indicated genotypes and treatment conditions. (**k**) Box plots quantifying peak Δ*F*/*F* values during SOCE in the indicated genotypes; **P<0.001, Mann–Whitney *U*-test; NS, not significant.

Mammalian septins, SEPT2, SEPT4 and SEPT5, belong to the SEPT2 class of septins^26^,^29^, and are positive regulators of SOCE in HeLa and T cells^25^. Because dSEPT7 is also a constituent of *Drosophila* septin complexes containing dSEPT1 (ref. 33), a *Drosophila* septin of the SEPT2 group (Table 1; Fig. 3a), we tested whether positive regulation of SOCE by septins of the SEPT2 subgroup is conserved between mammalian cells and *Drosophila* neurons.

### SEPT2 subgroup and dSEPT7 functions appear distinct

Simultaneous knockdown of *dSEPT1* (*Sep1* (refs 33, 34)) and *dSEPT4* (*Sep4* (refs 28, 35)), homologues of mammalian SEPT1 and SEPT4 in flies^29^ (Table 1), significantly attenuated SOCE in neurons (Fig. 3b–d) with resulting flight deficits in adult flies (Supplementary Fig. 3a). Thus, septins of the SEPT2 subgroup, which nucleate the septin complex and filament formation^36^ (Fig. 3a), function as positive regulators of SOCE in both vertebrates and *Drosophila.* This is in contrast to dSEPT7, which functions as a negative regulator of SOCE in *Drosophila* neurons (Fig. 2).

Conservation of septin function in SOCE was further tested in *Drosophila* neurons by application of forchlorfenuron (FCF), a pharmacological agent that perturbs septin filament organization and dynamics^37^,^38^, and leads to the loss of SOCE in mammalian cells^25^. Aggregation of Sep2-GFP^39^ was used as a cellular read-out for the efficacy of FCF treatment on inhibition of septin filament dynamics. Treatment of WT *Drosophila* neurons with 100 μM FCF for 45 min lead to Sep2-GFP aggregation (Fig. 3e) and a concurrent loss of SOCE (Fig. 3f,h,i), with no significant change in passive ER store release (Supplementary Fig. 3b), whereas FCF treatment for a shorter period of 20 min resulted in partial reduction of SOCE (Fig. 3f,h). These data support a requirement for septin filament dynamics during SOCE in *Drosophila* neurons, in agreement with their observed requirement for SOCE in non-excitable mammalian cells^25^. Interestingly, neurons with reduced dSEPT7 exhibit similar extent of Sep2-GFP aggregation as WT neurons (Fig. 3e), but normal SOCE after FCF treatment (Fig. 3g–i). In agreement with these data, SOCE in *itpr* mutant neurons with reduced dSEPT7 also remained unaffected on FCF treatment (Fig. 3j,k). Together, these data suggest that, although septin filament dynamics are required for activation of SOCE in *Drosophila* neurons, reduction of dSEPT7 overrides this requirement. The mechanism of dSEPT7 regulated Ca^2+^ uptake was investigated next.

### Ca^2+^ uptake in neurons with reduced dSEPT7 requires dOrai

**Figure 4:**
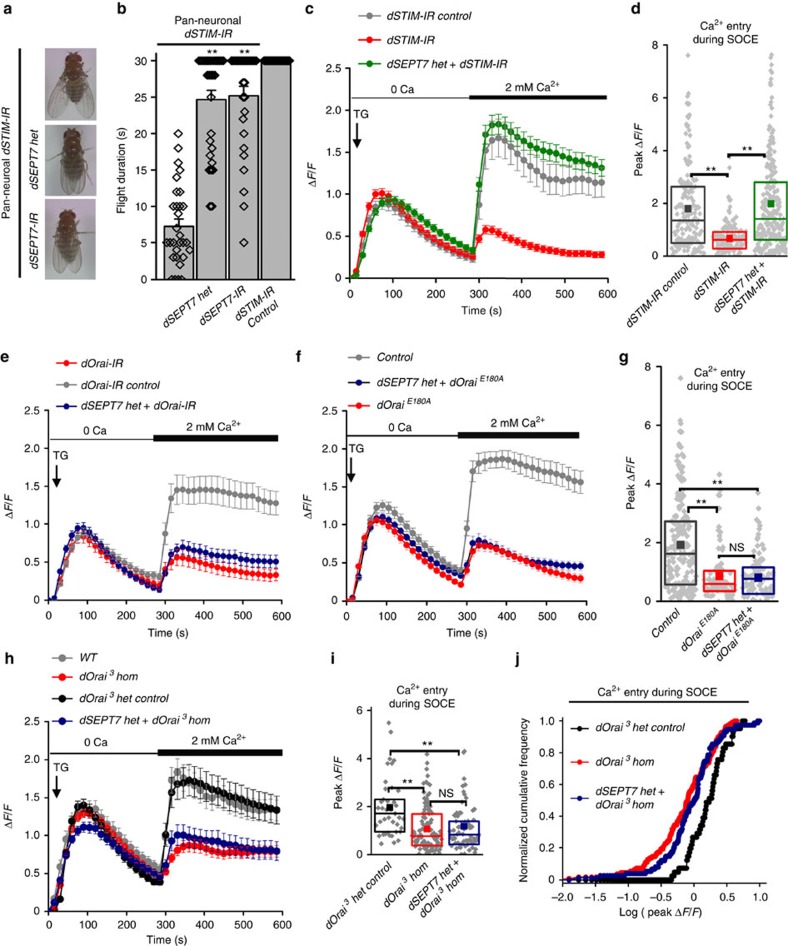
Extracellular Ca^2+^ entry obtained on dSEPT7 reduction requires functional dOrai channels. (**a**,**b**) Wing-posture defects (**a**) and reduced flight duration (**b**) in flies with pan-neuronal knockdown of dSTIM (*dSTIM-IR*) was rescued by dSEPT7 reduction with either a single copy of the deficiency allele or RNAi-mediated targeted knockdown in neurons*. N*≥30; **P<0.01, Mann–Whitney *U*-test; flight duration of *dSTIM* knockdown flies with reduced *dSEPT7* using either *dSEPT7 het* or *dSEPT7-IR* compared with that of *dSTIM* knockdown flies. (**c**) Reduced SOCE on *dSTIM* knockdown in primary neurons was restored on dSEPT7 reduction; *N*≥150 cells. Pan-neuronal *elav*^*C155*^*GAL4* was used to drive expression of *dSTIM-IR*. (**d**) Quantification of peak Δ*F*/*F* values obtained during SOCE (300–600 s) for the indicated genotypes; **P<0.001, Mann–Whitney *U*-test with Bonferroni correction. (**e**) Reduced SOCE obtained by knockdown of *dOrai* was not rescued on dSEPT7 reduction. Traces represent the mean (±s.e.m.) Δ*F*/*F* values obtained at each time point during TG-induced ER store Ca^2+^ depletion and SOCE for the indicated genotypes (*N*≥150 cells). Pan-neuronal *elav*^*C155*^ *GAL4* was used to drive expression of *dOrai-IR*. (**f**) Reduced SOCE obtained by expression of *dOrai*^*E180A*^ was not rescued by dSEPT7 reduction; *N*≥150 cells. Pan-neuronal *elav*^*C155*^*GAL4* was used to drive expression of *dOrai*^*E180A*^. Control refers to neurons with the *dOrai*^*E180A*^ transgenic construct, but no GAL4 driver, resulting in no expression of the transgene. (**g**) Box plots quantifying the peak Δ*F*/*F* values for SOCE (300–600 s) computed from the Ca^2+^ traces shown in **f** **P<0.001, Mann–Whitney *U*-test with Bonferroni correction. (**h**) Reduced SOCE obtained in neurons homozygous for a hypomorphic *dOrai* mutant allele (*dOrai*^*3*^ *hom* or *dOrai*^*3/3*^) was not rescued on dSEPT7 reduction. Traces represent the mean (±s.e.m.) Δ*F*/*F* values obtained at each time point during TG-induced ER store Ca^2+^ depletion and SOCE for the indicated genotypes (*N*≥150 cells). *dOrai*^*3*^ *het* control refers to neurons heterozygous for the *dOrai*^*3*^ hypomorphic allele (recessive). (**i**) Box plot quantifying the peak Δ*F*/*F* obtained during SOCE in the indicated genotypes. **P<0.001, Mann–Whitney *U*-test. (**j**) K–S plot analysing the peak Δ*F*/*F* obtained during SOCE in the indicated genotypes. *P*<0.001, K–S test.

Flies with pan-neuronal knockdown of *dSTIM* exhibit wing-posture and flight deficits^21^, similar to adult phenotypes of *itpr* mutants (Fig. 4a,b). dSEPT7 reduction either by genetic deletion or RNAi-mediated knockdown rescued both phenotypes in *dSTIM* knockdown flies (Fig. 4a,b). dSEPT7 reduction also restored robust extracellular Ca^2+^ uptake in neurons with dSTIM knockdown after store depletion (Fig. 4c,d; Supplementary Fig. 4a). This rescue was not accompanied by a concomitant increase of dSTIM protein in the CNS (Supplementary Fig. 4b), indicating that the rescue was not by upregulation of dSTIM levels. Reduction of dSEPT7, however, failed to improve Ca^2+^ entry in neurons with either reduced levels of dOrai or compromised dOrai function. Neurons cultured from animals with RNAi-mediated knockdown of *dOrai* (Fig. 4e; Supplementary Fig. 4c) and from a hypomorphic *dOrai* mutant (*dOrai*^*3/3*^ or *dOrai*^*3*^ *hom*; Fig. 4h) exhibit significantly reduced SOCE and this remained low on reducing dSEPT7 (Fig. 4e,h–j; Supplementary Fig. 4c). Similarly, reduction of dSEPT7 did not restore SOCE in neurons with overexpression of a mutant dOrai^E180A^, which functions as a dominant-negative *in vivo*^40^ (Fig. 4f,g). In agreement with this, dSEPT7 reduction also failed to improve flight in flies with pan-neuronal knockdown of dOrai (Supplementary Fig. 4d). Together, these data suggest that Ca^2+^ entry on reduction of dSEPT7 requires functional dOrai channels. Moreover, reduced dSEPT7 can compensate for reduced levels of the ER Ca^2+^ sensor dSTIM.

### dSEPT7 reduction leads to reorganization of dSTIM and dOrai

**Figure 5:**
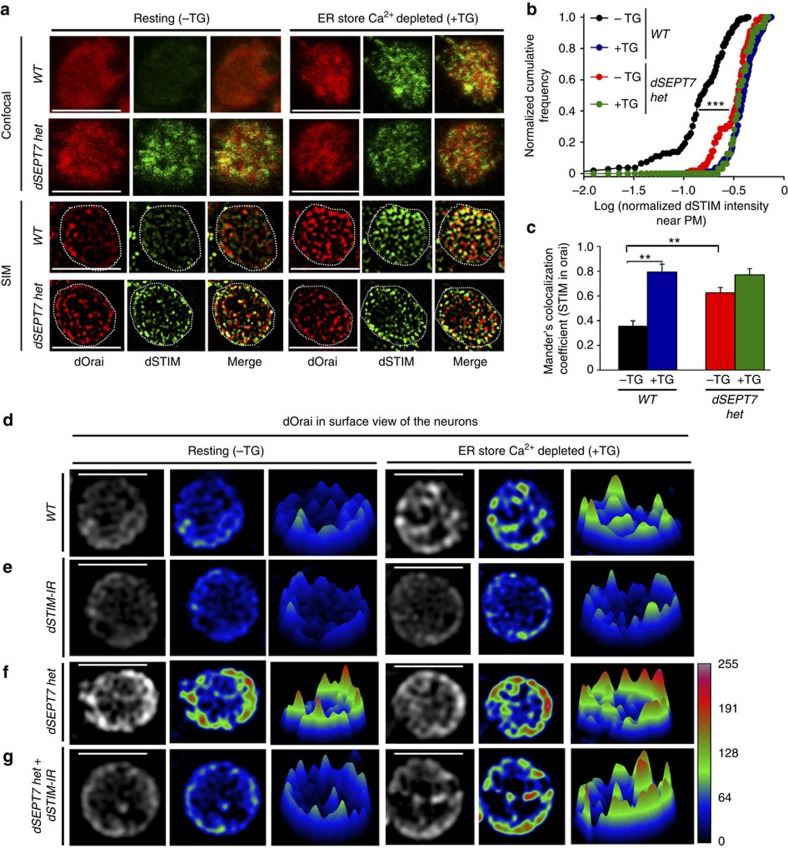
dSEPT7 reduction results in reorganization of dSTIM and dOrai in resting neurons. (**a**) Representative images of immunostained endogenous dSTIM and dOrai, in an optical section of a surface view of the PM and subcellular regions in close proximity of the PM, in either resting (−TG) or store-depleted (+TG) cells of the indicated genotypes. Scale bar, 5 μM. Images shown in the top two panels were acquired using confocal microscopy and those in the bottom two panels were acquired using structured illumination microscopy (SIM). (**b**) A K–S plot comparing normalized dSTIM intensities near the PM in the indicated genotypes either in resting conditions or after TG treatment. The distributions for TG-treated wild-type cells is shifted significantly to the right when compared with the resting cells, indicating a greater proportion of cells with higher normalized dSTIM intensities. The distribution for resting cells with reduced dSEPT7 is shifted significantly to the right when compared with the resting wild-type cells, indicating a higher proportion of cells with higher normalized dSTIM intensities. ***P<0.0001, K–S test, WT (−TG) compared with either WT (+TG) or *dSEPT7 het* (−TG). Normalized dSTIM intensity near the surface=dSTIM intensity in the optical section representing the cell surface/total dSTIM intensity for the cell. Images acquired using confocal microscopy were used for the analysis in **b**,**c**. (**c**) Bar graph quantifying the Mander’s co-localization coefficients for the amount of STIM intensity co-localizing with Orai intensity in the indicated genotypes and treatment conditions. **P<0.001, Mann–Whitney U test with Bonferroni correction. (**d**–**g**) Representative images of dOrai protein organization on the surface of resting and TG-treated cells of the indicated genotypes. dOrai was visualized by optimal image acquisition settings with confocal microscopy. Original grey scale images were deconvoluted and pseudo-coloured for better representation of high and low dOrai intensities with warmer colours representing higher intensities. A surface plot depicting the spatial distribution of dOrai intensities accompanies each image. Calibration bar represents the grey scale intensities corresponding to each colour. Scale bar, 5 μm.

To begin understanding how lower levels of dSEPT7 reduce the dependence of dOrai channel opening on dSTIM, we analysed the extent of endogenous dSTIM co-localization with dOrai near the PM in WT and *dSEPT7 het* neurons, using previously validated antibodies (Supplementary Fig. 5a,b)^40^,^41^. For this purpose, optical sections of ∼300 nm, identified by dOrai immunostaining and representing the PM and subcellular regions in its close proximity, were selected, and dSTIM fluorescence in these sections was quantified (Fig. 5a, confocal). Normalized dSTIM fluorescence detected in these optical sections was significantly higher in store-depleted cells when compared with the resting untreated cells (Fig. 5a, upper panels; Fig. 5b). This resulted in increased co-localization of dSTIM with dOrai after TG treatment (Fig. 5a, +TG, and Fig. 5c). Interestingly, significantly higher intensity of dSTIM was detected near the PM in resting neurons with reduced dSEPT7, and this PM localization was not enhanced significantly after store depletion (Fig. 5a, confocal—lower panels, Fig. 5b,c). Recruitment of STIM to the peripheral ER after store depletion is generally accompanied by clustering and has been visualized by expression of tagged STIM constructs in non-excitable cells^3^,^12^. In WT *Drosophila* neurons, dSTIM near the PM appeared mildly clustered after store depletion (Fig. 5a, confocal, +TG). These clusters were better resolved and visualized by structured illumination microscopy (SIM) in store-depleted neurons as well as in *dSEPT7 het* neurons at rest and after store depletion (Fig. 5a, SIM).

The appearance of dOrai clusters concomitant with dSTIM was investigated next. A majority of WT *Drosophila* neurons (∼65%) exhibit diffuse distribution of endogenous dOrai proteins at the PM at rest (Fig. 5a, resting). This diffuse distribution changed to visibly distinct high-intensity clusters in ER store-depleted neurons treated with TG (Fig. 5a, +TG and Fig. 5d, +TG). dOrai clusters, obtained after store depletion by TG, were also observed through SIM (Fig. 5a, SIM). In a fraction (∼35%) of resting WT neurons, dOrai clusters were observed (Fig. 5d, resting), but their normalized intensity was significantly lower as compared with dOrai clusters observed in store-depleted neurons (Supplementary Fig. 6b,c,f). As with dSTIM, resting neurons with reduced dSEPT7, exhibit greater numbers of high-intensity dOrai clusters, and the intensity of these clusters did not change significantly on store depletion (Fig. 5f; Supplementary Fig. 6b,c,g). Thus, reduced dSEPT7 levels pre-dispose resting neurons towards a state that could allow spontaneous Ca^2+^ entry through dOrai. Higher-resolution SIM images confirmed these observations, where, in resting neurons with reduced dSEPT7, dOrai clusters co-localized with visibly distinct dSTIM clusters (Fig. 5a, SIM).

In agreement with previous observations in non-excitable cells^12^, the intensity of dOrai clusters was significantly lower in dSTIM knockdown cells as compared with WT cells after store depletion (Fig. 5e; Supplementary Fig. 6b,d). dSTIM knockdown also resulted in significant reduction of high-intensity dOrai clusters in resting neurons with reduced dSEPT7, suggesting that clustering of dOrai in resting neurons requires dSTIM (Fig. 5g; Supplementary Fig. 6b).

**Figure 6:**
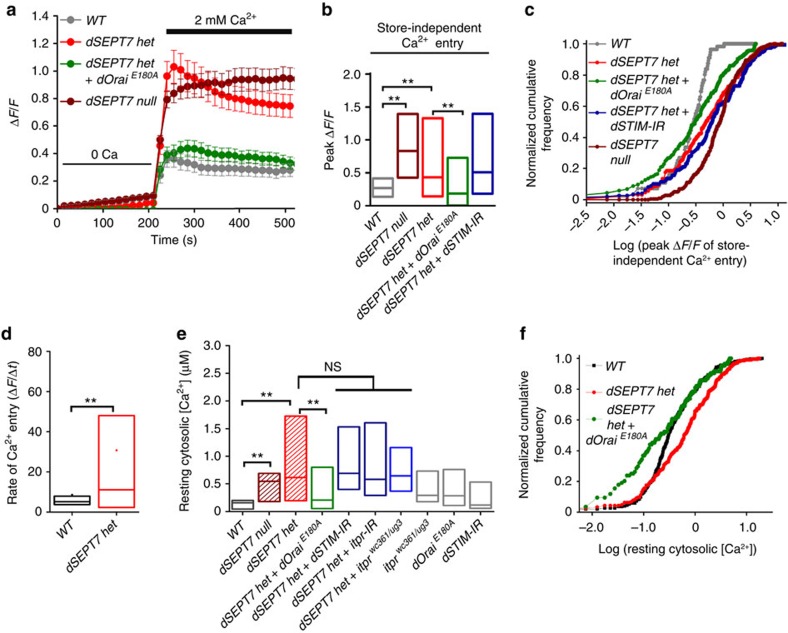
dSEPT7 reduction results in a store-independent activation of dOrai channels in resting neurons. (**a**) Changes in cytosolic Ca^2+^ monitored as Δ*F*/*F* values during store-independent Ca^2+^ entry on Ca^2+^ add-back, after monitoring basal Ca^2+^ fluctuations in cells plated in ‘0 Ca medium’ for 225 s. Store-independent Ca^2+^ entry in neurons with reduced (*dSEPT7 het*) or no dSEPT7 (*dSEPT7 null*) is higher when compared with WT neurons, and is significantly reduced on expression of *dOrai*^*E180A*^ (*N*≥150 cells). (**b**) Box plot quantifying the peak Δ*F*/*F* obtained during store-independent Ca^2+^ entry (200–500 s) in the indicated genotypes. **P<0.01, Mann–Whitney *U*-test with Bonferroni correction. (**c**) K–S plot analysing the peak Δ*F*/*F* obtained during Ca^2+^ entry in the indicated genotypes. The distribution for neurons with reduced (*dSEPT7 het*) or no dSEPT7 (*dSEPT7 null*) is significantly shifted to the right compared with wild-type neurons, indicating a higher proportion of cells with higher peak Δ*F*/*F* values. The distribution for *dSEPT7 het* cells is significantly shifted to the left after expression of dOrai^E180A^, indicating a greater proportion of cells with lower peak Δ*F*/*F* values. *P*<0.001, K–S test for ‘*dSEPT7 het*’ or ‘*dSEPT7 null*’ compared with WT and ‘*dSEPT7 het*’ compared with ‘*dSEPT7 het+dOrai*^*E180A*^’. (**d**) Rate of Ca^2+^ entry in the indicated genotypes. Rate of Ca^2+^ entry expressed as Δ*F*/Δ*t*, where Δ*F*=*F*_255_−*F*_225_ and Δ*t*=30 s. **P<0.01, Mann-Whitney U test. (**e**) Resting cytosolic [Ca^2+^] in the indicated genotypes measured using Indo-1AM (Methods). Higher resting cytosolic [Ca^2+^] in neurons with reduced or no dSEPT7 (median ∼500 nM) is restored to WT levels (median ∼200 nM) on expression of *dOrai*^*E180A*^. The horizontal line in the middle of the box represents the median. **P<0.001, Mann–Whitney *U*-test with Bonferroni correction. (**f**) K–S plots analysing the distribution of resting cytosolic [Ca^2+^] in the indicated genotypes. The distribution for neurons with reduced dSEPT7 is significantly shifted to the right compared with WT, indicating a greater proportion of cells with higher resting cytosolic [Ca^2+^]. This distribution was significantly shifted to the left on expression of *dOrai*^*E180A*^, indicating a higher proportion of cells with lower resting cytosolic [Ca^2+^]; *P*<0.001, K–S test.

The high levels of dSTIM present near the PM and the resulting dOrai clusters observed in resting neurons with reduced dSEPT7 prompted us to investigate their ability to support Ca^2+^ entry in the absence of store depletion. WT neurons exhibit a low level of spontaneous Ca^2+^ entry at rest on addition of Ca^2+^ to the extracellular medium (Fig. 6a). In *dSEPT7 het* and *dSEPT7 null* neurons, spontaneous uptake of extracellular Ca^2+^ was significantly higher as compared with WT neurons (Fig. 6a–c). Importantly, higher uptake of extracellular Ca^2+^ by neurons with reduced dSEPT7 was significantly reduced on expression of the ‘dominant-negative’ *dOrai*^*E180A*^ transgene (Fig. 6a–c). In agreement with the rescue of flight deficits of dSTIM knockdown animals by introducing *dSEPT7 het*, store-independent Ca^2+^ entry remained high in *dSEPT7 het+dSTIM-IR* neurons (Fig. 6b,c; see discussion). The rate of Ca^2+^ uptake was significantly higher in *dSEPT7 het* neurons when compared with WT (Fig. 6d).

A predicted outcome of such constitutive Ca^2+^ entry would be higher basal cytosolic Ca^2+^ levels in neurons with reduced dSEPT7. Indeed, resting cytosolic [Ca^2+^] was higher in *dSEPT7 het* and *dSEPT7 null* neurons when compared with WT neurons (Fig. 6e,f). Expression of *dOrai*^*E180A*^ in *dSEPT7 het* neurons significantly reduced resting cytosolic [Ca^2+^] to levels comparable to WT neurons (Fig. 6e,f). Thus, reduction of dSEPT7 promotes Ca^2+^ entry through dOrai in resting neurons, which is independent of ER store depletion.

dOrai clusters were also reduced in neurons with IP_3_R knockdown after store depletion (Supplementary Fig. 6a,b,e), suggesting a role for the IP_3_R in dSTIM-mediated clustering and activation of dOrai. However, knockdown of the IP_3_R did not reduce dOrai clusters observed in *dSEPT7 het* neurons at rest (Supplementary Fig. 6a,b). Resting cytosolic [Ca^2+^] also remained high in neurons with reduced dSEPT7 after either IP_3_R knockdown or dSTIM knockdown (Fig. 6e) and in IP_3_R mutant neurons with reduced dSEPT7 (Fig. 6e, *dSEPT7 het*+*itpr*^*wc361/ug3*^). These data are consistent with the observation that dSEPT7 reduction allowed extracellular Ca^2+^ uptake in neurons with either reduced IP_3_R (Fig. 2f) or dSTIM (Fig. 4c), but not dOrai (Fig. 4e) and support a model where higher cytosolic [Ca^2+^] in neurons with dSEPT7 reduction results from store-independent Ca^2+^ entry through dOrai.

The mechanism by which dSEPT7 supports greater dSTIM localization to the PM and Orai channel opening in the absence of store Ca^2+^ depletion needs further elucidation. In preliminary experiments, we observed a fraction of endogenous dSEPT7 closely apposed to the ER, marked by GFP-tagged protein disulphide isomerase (Supplementary Fig. 5c, d, upper panels) and in an optical section of the cell surface where it co-localized with the cortical ER, (Supplementary Fig. 5d, lower panels). In addition, dSEPT7 and dOrai co-localized at the periphery of neuronal cell bodies (Supplementary Fig. 5e, upper panels) as well as in neuronal projections (Supplementary Fig. 5e, lower panels; arrows), in agreement with similar PM association found for SEPT7 in mammalian neurons^42^,^43^. The intracellular distribution of dSEPT7 near the ER and in the proximity of the PM suggests a direct role in the organization and function of dSTIM and dOrai.

## Discussion

In this study, we show that dSEPT7 prevents dOrai-mediated spontaneous Ca^2+^ entry in neurons. Consequently, lower dSEPT7 levels lead to Ca^2+^ store-independent constitutive opening of dOrai channels in resting neurons. Thus, dSEPT7 functions as a ‘molecular brake’ on dOrai channel activation in unstimulated neurons. Consistent with the negative regulatory function, dSEPT7 overexpression resulted in lower SOCE in neurons (Fig. 2h).

**Figure 7:**
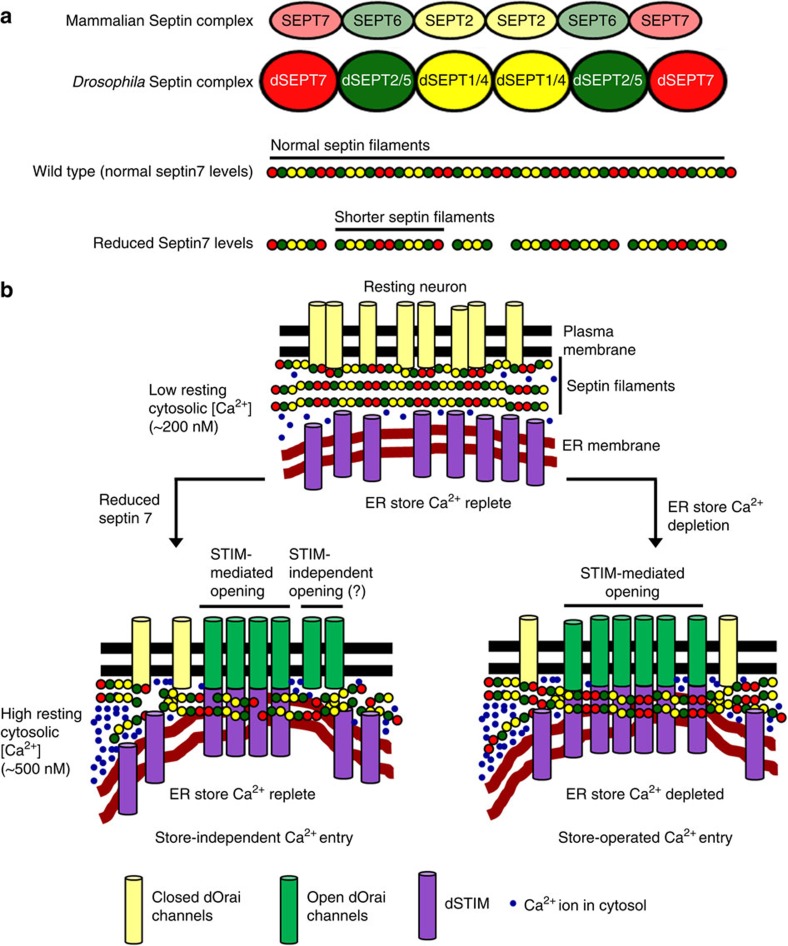
A model explaining activation of Orai upon dSEPT7 reduction in neurons. (**a**) Arrangement of mammalian and *Drosophila* septins in septin complexes (hetero-hexamers) that are the basic building blocks of septin filaments. Reduction of dSEPT7 is predicted to result in breaks in the linear septin filaments present in wild-type cells, leading to the formation of shorter septin filaments. (**b**) Septin filaments closely associate with the PM and near the ER in resting neurons. ER store Ca^2+^ depletion results in a reorganization of these filaments, followed by movement of STIM to the peripheral ER, dSTIM/dOrai coupling and Ca^2+^ entry through dOrai (SOCE). dSEPT7 reduction leads to formation of shorter septin filaments that support dSTIM recruitment to the peripheral ER in resting neurons and promote Orai channel opening. This store-independent activation of dOrai results in elevation of cytosolic [Ca^2+^].

dSEPT7 forms a complex with dSEPT1 and dSEPT2 (ref. 33) similar to mammalian SEPT7, which forms linear hexamers with SEPT2 and SEPT6 (ref. 44; Fig. 7a). SEPT7 occupies terminal positions in this hexamer^44^. These hexamers are further linked to form non-polar linear septin filaments^26^,^45^ (Fig. 7a). We propose a model where dSEPT7-containing septin filaments, in resting neurons with normal dSEPT7 levels, maintain a ‘check’ on dSTIM recruitment to the ER–PM regions (Fig. 7b, resting neuron). Reducing dSEPT7 removes this block in neurons with replete stores and results in dSTIM recruitment to the ER–PM regions, with concomitant formation of dOrai clusters and opening of Orai channels (Fig. 7b, reduced Septin 7). STIM recruitment to the peripheral ER followed by clustering of Orai has been visualized by expression of tagged molecules in most cell types after store depletion^12^,^25^. However, Orai clusters are not necessary for opening of Orai channels^12^,^46^,^47^. In agreement with these previous findings from non-excitable cells, lower dSTIM levels also reduce dOrai clusters in resting neurons with reduced dSEPT7 (Fig. 5g; Supplementary Fig. 6b). Higher spontaneous Ca^2+^ entry and basal cytosolic [Ca^2+^] in *dSEPT7 het* neurons with *dSTIM* knockdown (Fig. 6b,c) suggest the presence of non-clustered but active dOrai channels, and indicate that dSEPT7-containing septin filaments help in organizing membrane domains that favour the inactive state of dOrai channels. Reducing dSEPT7 possibly leads to the presence of shorter septin filaments (Fig. 7a,b), which we speculate help reorganize PIP_2_ in the PM^25^ and formation of membrane domains^48^,^49^ that favour dOrai channel opening by residual dSTIM proteins. Thus, reduction of dSEPT7 at the PM probably mimics a membrane state that is triggered by septin filament reorganization after store depletion and is permissive for Orai activation (Fig. 7b).

Depletion of ER store Ca^2+^ is the primary stimulus for STIM translocation to the ER–PM regions^3^, although recent findings indicate multiple other proteins additionally regulate this process^50^,^51^. Overexpression of one such regulator, STIMATE resulted in STIM clustering and recruitment to the ER–PM regions in mammalian cells with replete ER store Ca^2+^ levels^50^. Importantly, it also resulted in store-independent Ca^2+^ entry through Orai channels^50^. In neurons with reduced dSEPT7, we observed increased localization of dSTIM clusters near the ER–PM regions (Fig. 5a,b). These dSTIM clusters may arise due to a small drop in ER calcium, undetected by TG-mediated passive store release in our experiments. Because partial depletion of dSTIM with an RNAi continued to support store-independent Ca^2+^ entry and higher resting cytosolic [Ca^2+^] in *dSEPT7 het* neurons to the same extent as *dSEPT7 het* neurons with WT levels of dSTIM (Fig. 6c,e), we predict that Orai-mediated Ca^2+^ entry observed on dSEPT7 reduction maybe a cumulative effect of dOrai activation by residual dSTIM proteins and other non-canonical modes of Orai activation, which remain to be elucidated.

Septins are ubiquitous proteins, but the levels of individual septin subunits and their functions vary across cell types^26^. For example, dSEPT7 can function differently during cytokinesis and cellularization, based on the cell type under study^28^. Interestingly, the role of mammalian SEPT7 in cytokinesis is also cell-type specific^52^. Thus, the functional consequences of modulating septin levels in cells depends on the subgroup of septin depleted, its effect on septin filament organization and ultimately the cell-type under investigation. In this context, our findings that the SEPT2 subgroup of septins (Table 1; Fig. 7a) function as positive regulators of SOCE, whereas dSEPT7 acts as a ‘molecular brake’ on Orai activation, are not unprecedented. The regulation of Orai function by septins will require a better understanding of the nature of septin complexes and filaments present in resting neurons and in neurons after store depletion.

SOCE is required in neurons of the *Drosophila* flight circuit for transcriptional regulation of genes required during maturation of the flight circuit^40^. We have earlier demonstrated that raising cytosolic [Ca^2+^] by reducing sarco-endoplasmic reticulum Ca^2+^ATPase function with a dominant-negative mutant allele *CaP60A*^*kum170/+*^ (ref. 53), partially restores flight bouts in *itpr* mutants^54^, and the length of these flight bouts are further enhanced by introducing dominant hypermorphic alleles of *dOrai*^21^. A similar increase in resting cytosolic [Ca^2+^] is observed in *Drosophila* neurons with reduced dSEPT7; this is very likely responsible for the rescue of *itpr* mutant flight deficits obtained on dSEPT7 reduction. Thus, Septin 7 functions as a novel regulator of neuronal Ca^2+^ homeostasis with an impact on physiological and behavioural phenotypes.

The ability of SEPT7 to control cytosolic Ca^2+^ levels in *Drosophila* neurons through Orai needs testing in mammalian neurons because deranged Ca^2+^ homeostasis has been linked to several neurodegenerative diseases^55^,^56^. Our findings could thus provide insights into new therapeutic targets for regulating neuronal Ca^2+^ homeostasis.

## Methods

### Fly strains

All flies were reared on corn flour agar medium supplemented with yeast at 25 °C. Canton-S was used as the WT strain. Single point mutations in the *Drosophila itpr* gene have been described earlier^30^. The genetic deficiency allele *pnut*^*xp*^^27^(BL5687) and pan-neuronal *elav*^*C155*^*GAL4* (BL458) were obtained from the Bloomington Stock Centre, Indiana, USA. An RNAi line (referred to as ‘*itpr-IR*’) for the *itpr* gene (1063) was obtained from the National Institute of Genetics (NIG), Japan. RNAi lines for *dSTIM*(v47073), *dOrai*(v12221), *dSEPT7* (v11791), *dSEPT1* (v101445) and *dSEPT4* (v7742) were obtained from the Vienna Drosophila RNAi Centre (VDRC), Vienna, Austria. The RNAi strains obtained from VDRC and NIG contain, short regions (∼200 bp) of the target gene cloned as inverted repeats. These inverted repeats are introduced as transgenes under control of the upstream activator sequence (UAS)^57^. When expressed in neurons using the pan-neuronal *elav*^*C155*^*GAL4*, a resulting hairpin RNA is formed *in vivo* and recognized by the existing RNAi machinery within the cell. This subsequently generates smaller double-stranded RNAs of 21–22 nucleotides in length, which efficiently target and knockdown messenger RNAs from the gene of interest. Gene sequences, cloned as inverted repeats for each gene, are publicly available from the VDRC (http://stockcenter.vdrc.at/control/main) and NIG (www.nig.ac.jp/nig/) stock centres. The hypomorphic *dOrai* allele (*dOrai*^*3*^)^40^, *UASdSTIM* (referred to as *dSTIM*^*+*^)^22^, *UASdOrai*^*E180A*^ (referred to as *dOrai*^*E180A*^)^40^ and a transgenic strain expressing Sep2-GFP under the control of the endogenous *Sep2* promoter^39^ have been described earlier. A full-length complementary DNA of the *dSEPT7* (or *pnut*) gene (BDGP LD37170 (ref. 58)) was subcloned into the *pUASTattB* vector between the EcoR1 and Xba1 sites, and microinjected into embryos to generate the *UASpnut*^*+*^(*UASdSEPT7*^*+*^) transgenic line. The pan-neuronal *elav*^*C155*^*GAL4* was used for expression of the gene-specific RNAi or the *dSEPT7*^*+*^ transgene in all neurons of the fly. ‘*Gene-IR-control*’ refers to animals containing the RNAi construct, but no GAL4, resulting in no expression of the RNAi. All fly strains were generated using standard protocols for fly genetics.

### Flight assays

Flight assays were performed as described earlier^40^. In brief, single flies were aged post-eclosion for 3 days, anaesthesized on ice for 15 min before recording flight durations. Each fly was tethered in the neck region between the head and thorax to a thin-metal wire. Flight was monitored for 30 s after delivery of a gentle mouth blown air-puff. Flight duration for each fly was recorded manually. Statistical significance between genotypes was computed with the Mann–Whitney *U*-test on the Origin 8.0 software (Micro Cal).

### Primary neuronal culture

Primary neurons were cultured from the third instar larval CNS (CNS comprising the brain and ventral ganglion) as described previously^21^,^40^. In brief, larval CNS was dissected and subjected to enzymatic digestion using 0.75 μg μl^−1^ collagenase and 0.40 μg μl^−1^ dispase for ∼40 min. Single dissociated neurons were spun down, followed by plating in growth medium comprising of DMEM/F12-1065 (Gibco) supplemented with 20 mM HEPES (pH 7.2), 50 U ml^−1^ penicillin (Invitrogen), 50 μg ml^−1^ streptomycin (Invitrogen) and 10 μg ml^−1^ Amphotericin B (Invitrogen). Cells were incubated at 25 °C with 5% CO_2_ for 14–16 h. All chemicals for cell culture were obtained from Sigma-Aldrich unless mentioned otherwise. This protocol primarily supports growth of neuronal cells, and the glial cells remain at <1% of the total cell population^59^.

### Calcium imaging in primary neurons

For the measurement of store Ca^2+^ release and SOCE, cells were washed with M1 medium^21^, following which they were loaded with 2.5 μM of Fluo-4 acetoxymethylester (AM; Life Technologies) with 0.002% Pluronic-F-127 (Sigma-Aldrich) in M1 medium for 30 min in the dark. Following dye loading, cells were washed in M1 and placed in nominally ‘0’ Ca^2+^ M1 (containing 12 nM-free Ca^2+^) supplemented with 0.5 mM EGTA and imaged for Ca^2+^ changes. Imaging and quantification were performed as described previously^21^. Images were acquired every 15 s (for passive store depletion induced SOCE) or every 10 s (for CCh-stimulated SOCE) for a total duration of 10 min (600 s) on a Nikon TE2000 inverted wide-field microscope equipped with × 60/1.4 numerical aperture (NA; oil) objective lens, using the 488-nm excitation and 520-nm emission filter sets. Store depletion was induced by adding 10 μM TG (Life Technologies) or 20 μM Carbamoylcholine chloride (CCh; Sigma) after the first frame of acquisition. CCh-containing medium was exchanged with ‘0 Ca’ medium after 200 s, and the cells were imaged for another 100 s before Ca^2+^ add-back. CaCl_2_ was added after the 20th frame of acquisition (or 300 s). CCh measurements were performed with overexpression of muscarinic acetylcholine receptor in neurons of all genotypes. For the measurement of resting cytosolic Ca^2+^, a previously described protocol^21^ was followed. In brief, cells were incubated with 5 μM Indo-1 AM and 0.002% Pluronic F-127 in M1 media for 45 min at room temperature in the dark. Cells were washed twice with M1 before and after dye incubation and finally covered with M1 for imaging. Data acquisition was performed by 365-nm excitation and 410/485 dual-emission filter sets at 5-s interval for five frames, after which 10 μM ionomycin (Calbiochem, USA) was added to obtain the maximum florescence intensity and imaged for 10 more frames. EGTA (1 mM) and 0.01% Triton-X-100 were added after the 10th frame to obtain the minimum florescence intensity for each cell. Image acquisition for basal calcium measurements was performed on an Olympus IX81-ZDC2 inverted wide-field microscope equipped with focus drift compensating and × 20/0.5 NA (air), × 60/1.35 NA (oil) and × 100/1.4 NA (oil) objective lenses. Excitation of fluorescent Ca^2+^ indicator dye was performed using specific wavelength illumination from a halogen arc lamp with a TILL Polychrome 5000 monochromator (TILL Photonics, Graefelfing, Germany) for variable bandwidth and intensity. Emitted light was detected through band-pass filter sets (Chroma, Brattleboro, VT). Image acquisition was performed using the Andor iXON 897E EMCCD camera and AndoriQ 2.4.2 imaging software. The time-lapse acquisition mode of the software was used to follow fluorescence changes over time.

### Pharmacological perturbation of septin dynamics

For perturbation of septin dynamics, 14–16-h-old primary neurons were washed with M1 medium, following which they were covered with growth medium (DMEM supplemented with 20 mM HEPES and antibiotics) containing either dimethylsulphoxide (0.1%, solvent control) or 100 μM FCF (Sigma-Aldrich) and incubated at room temperature for either 20 or 45 min. This was followed by three consecutive washes with M1 medium, after which the cells were loaded with Fluo-4-AM for 30 min for calcium measurements or fixed for immunostaining.

### Data analysis

For quantifying the changes in fluorescence, images were processed using the Image Pro plus software, V1.33 (Media Cybernetics). Region of interest (ROI) was drawn around each cell, and the fluorescence intensity at each time points was determined and plotted using the Origin 8.0 software as follows: Δ*F*/*F*=(*F*_*t*_−*F*_basal_)/*F*_basal_ for each time point, where *F*_*t*_ is fluorescence at the time point and *F*_basal_ is the fluorescence of the cell when starting the experiment. Peak values of Δ*F*/*F* were obtained for every cell, and a box chart representing the data spread was plotted. The rectangular boxes represent the spread of data points between 25 and 75% of cells and the horizontal line within each box is the median. The smaller box inside each box represents the mean. Significance was computed for different data sets using Mann–Whitney *U*-test for nonparametric distributions. The distribution of peak Δ*F*/*F* values or resting cytosolic [Ca^2+^] for different genotypes and conditions were also analysed using Kolmogorov–Smirnov (K–S) test. For K–S tests, the cumulative frequency of the peak Δ*F*/*F* values or resting cytosolic [Ca^2+^] was normalized to the total number of cells and plotted against the log of the peak Δ*F*/*F* values or resting cytosolic [Ca^2+^]. Significance was computed based on the maximum difference between the two distributions. Rate of Ca^2+^ entry during SOCE was calculated by computing the rate of change in fluorescence intensity (Δ*F*) between the 315 and 285 s time points and expressed as Δ*F*/Δ*t*, where Δ*t*=30 s.

For computation of resting cytosolic [Ca^2+^], fluorescence values obtained at each time point were converted to ratios of 410/485 emission. The absolute concentration of free calcium was calculated using the Grynkiewicz equation: [Ca^2+^]_i_ (μM)=*K*_d_ × (*F*_basal_−*F*_min_)/(*F*_max_−*F*_basal_), where *F*_basal_ is the basal cytosolic fluorescence (at the first frame or start of imaging), *F*_max_ is the peak fluorescence obtained after adding 10 μM ionomycin (to allow the cytosolic Ca^2+^ to equilibrate to external Ca^2+^) and *F*_min_ refers to the fluorescence on chelating all free cytosolic Ca^2+^ with EGTA. The published *K*_d_ value of 1.16 μM for Indo-1 in *Drosophila* cells was used^60^.

### Immunocytochemistry and confocal microscopy

Primary neuronal cultures, with or without TG treatment for 5 min, were washed with PBS (pH=7.5), followed by fixation with 3.5% paraformaldehyde in PBS for 20 min at room temperature. Fixed neurons were washed once with a wash buffer (1/10th dilution of blocking buffer) and permeabilized with three 10-min washes of wash buffer, followed by blocking with blocking buffer (5% bovine serum albumin (w/v), 0.5% Triton X (v/v), 0.5% glycerol (v/v) in PBS) for 1 h. The cultures were incubated overnight with primary antibodies at the appropriate dilution in wash buffer. The following day cells were washed three times, for 10 min each, with wash buffer at 4 °C. Incubation with the appropriate secondary antibody was in wash buffer for 30 min at 4 °C, 45 r.p.m., followed by three 15-min washes with wash buffer at room temperature. Cells were finally covered with 60% glycerol (v/v) for imaging in an inverted Olympus FV1000 confocal microscope. Images were acquired through a × 60/oil objective with × 6 optical zoom, with the Fluoview 2.1 C software and FV10-SPD detectors, under optimal settings. Entire cell was imaged using several sequential optical sections of 300-nm thickness. For immunostaining of Sep2-GFP, rabbit anti-GFP antibody (1:10,000; catalogue no. A6455, Life Technologies) was used. dSEPT7 (or Pnut) was detected with a mouse anti-Pnut antibody^27^ (1:4; catalogue no. 4C9H4, DSHB, University of Iowa, Iowa). A dOrai-specific antibody generated in rats was used at a dilution of 1:1,000 and has been described earlier^40^. Two anti-dSTIM mouse antibodies^41^ (8G1) and (3C1) mixed 1:1 were used at a dilution of 1:20. Secondary antibodies used at a dilution of 1:400 are as follows: anti-rabbit Alexa Fluor 488 (catalogue no. A1108; Life Technologies), anti-mouse Alexa Fluor 568 (catalogue no. A1104; Life Technologies) and anti-rat Alexa Fluor 633 (catalogue no. A21094; Life Technologies).

### Particle intensity analysis

For the analysis of dOrai particle intensities, raw confocal images (grey scale) were exported as TIFF files. A ROI was drawn around each cell and a mask image was generated using ROIs for all cells in a field. Subsequent analysis of the intensity of each particle was carried out in Matlab R2012 (Licence number 828962; host ID: A4BADB34D85C). The raw particle intensities were normalized to the mean intensity of dOrai for each cell to account for slight variations in antibody staining. The normalized dOrai particle intensities are quantified for each genotype and treatment condition (Supplementary Fig. 6b). Normalized intensities of particles between 3 and 8 pixels were used for analysis. Statistical significance was computed using the Mann–Whitney *U*-test and the K–S test. For representation, images were deconvoluted with the deconvolve three-dimensional (3D) plugin^61^ of ImageJ 1.41o (National Institute of Health, USA)^62^. The diffraction PSF 3D plugin in ImageJ was used to generate a theoretical point spread function (PSF) that was next used in the ‘Deconvolve 3D’ plugin to generate the deconvoluted image with a single iteration. The deconvoluted image was pseudo-coloured and a surface plot generated using ImageJ. For analysing the extent of co-localization, Pearson’s Correlation Coefficients and Mander’s co-localization coefficients were calculated using ImageJ.

### Quantification of STIM intensities near the surface

Untreated or TG-treated cells were fixed and the endogenous STIM and Orai proteins labelled using the respective antibodies in the same cell. Confocal images were obtained using simultaneous acquisition in both channels (STIM, excitation wavelength: 488 nm and Orai, excitation wavelength, 633 nm), using an Olympus FV1000 Confocal microscope as described earlier. For computation of STIM intensities near the PM, an optical section that represents the PM and intensities approximately within 300 nm from the coverslip were selected using immunostaining against Orai. The same optical section was selected from the other channel (showing STIM immunostaining) and the STIM intensities in this optical section computed using ImageJ. This STIM intensity near the PM was normalized to the total STIM fluorescence for the whole cell and expressed as ‘normalized STIM fluorescence near the PM’. Total STIM intensity was not significantly different between resting and TG-treated cells. Single-stack images from each of these two channels (showing the PM Orai staining and STIM intensity near the PM) were used to analyse STIM/Orai co-localization using ImageJ.

### Structured illumination microscopy

SIM was performed on a Nikon N-SIM microscope with a × 100 (oil, NA 1.49) CFI SR APOCHROMAT objective using the 488- or 633-nm laser lines. Cell footprints were imaged as a single-plane image. The super-resolution image obtained using 3D SIM was reconstructed, using the Nikon NIS ELEMENT Software version 4.30. Sample preparation was similar to that used for confocal imaging. Optimum intensity settings were maintained to obtain the super-resolution images.

### Western blots

Larval CNS or adult heads of appropriate genotypes were dissected in cold dissection buffer (20 mM HEPES, 100 mM KCl, pH 7.5). Homogenization of 10 brains or adult heads was performed in 100 μl of homogenizing buffer (20 mM HEPES, 100 mM KCl, 1% Triton-X-100, 1 mM PMSF, pH 7.5) and 15 μl of the homogenate was run on a 6% SDS–polyacrylamide gel (for IP_3_R) or 8% SDS–polyacrylamide gel (for all other proteins). The protein was transferred to a nitrocellulose membrane by standard protocols, and the membrane was incubated in the primary antibody overnight at 4 °C. Primary antibodies were used at the following dilutions: rat anti-dOrai, 1:1,000; mouse anti-β-tubulin monoclonal, 1:5,000 (catalogue no. E7, DSHB); and mouse anti-Pnut, 1:20 (catalogue no. 4C9H4, DSHB). The affinity-purified anti-InsP_3_R rabbit polyclonal antibody^31^(IB-9075) was used at a dilution of 1:300. A mouse anti-spectrin antibody (1:50; catalogue no. 3A9, DSHB) was used as a loading control for IP_3_R. Two anti-dSTIM mouse antibodies (8G1) and (3C1) mixed 1:1 were used at a dilution of 1:20. Secondary antibodies used were anti-mouse HRP (1:3,000; catalogue no. 7076S, Cell Signaling Technologies), anti-rabbit HRP (1:3,000; catalogue no. 32260, Thermo scientific, Rockford) and anti-rat HRP (1:10,000; catalogue no. 012030003, Jackson Immuno Research). Proteins were detected on the blot by a chemiluminiscent detection solution, SuperSignal West Dura Extended Duration Substrate (catalogue no. 34075, Thermo Scientific). Full-size western blots are shown in Supplementary Fig. 7. Images of the blots shown in the main and Supplementary Figs have been cropped for presentation.

### Statistical tests

Unless otherwise mentioned, statistical significance was computed between a pair of genotypes or treatment conditions, indicated using brackets in the figures, with the Mann–Whitney *U*-test or K–S test. If multiple genotypes or treatment conditions were compared, the *P* value used for determining the significance of the differences observed was calculated after applying Bonferroni correction. Nonparametric tests were used for computation of statistical significance as most of the data are not normally distributed.

### Data availability

The data that support the findings of this study are available from the corresponding author on request.

## Additional information

**How to cite this article:** Deb, B. K. *et al*. Store-independent modulation of Ca^2+^ entry through Orai by Septin 7. *Nat. Commun.* 7:11751 doi: 10.1038/ncomms11751 (2016).

## Supplementary Material

Supplementary Information Supplementary Figures 1 - 7

## Figures and Tables

**Table 1:** Nomenclature of septin subunits in mammals and *Drosophila*.

Septin subgroups	SEPT2	SEPT6	SEPT7	SEPT3
*Homo sapiens*	SEPT1, SEPT2, SEPT4, SEPT5	SEPT6, SEPT8, SEPT10, SEPT11	SEPT7	SEPT3, SEPT9, SEPT12
*Drosophila melanogaster*	dSEPT1, dSEPT4	dSEPT2, dSEPT5	dSEPT7	No representatives
