# Publisher Correction: Store-independent modulation of Ca^2+^ entry through Orai by Septin 7

**DOI:** 10.1038/ncomms16230

**Published:** 2018-07-18

**Authors:** Bipan Kumar Deb, Trayambak Pathak, Gaiti Hasan

Nature Communications 7: Article number: 11751; DOI: 10.1038/ncomms11751 (2016); Published 05 26 2016, Updated 07 18 2018

The original HTML version of this Article had an incorrect article number of ‘0’; it should have been ‘11751’. This has now been corrected in the HTML version of the Article. The PDF version was correct from the time of publication.

