# Supplementary material for: Store-independent modulation of Ca2+ entry through Orai by Septin 7
Source: Nat Commun. 2016 May 26;7:11751. doi: 10.1038/ncomms11751 (PMC4894974; doi:10.1038/ncomms11751)
Supplement: Supplementary Information — Supplementary Figures 1 - 7 [file ncomms11751-s1.pdf]

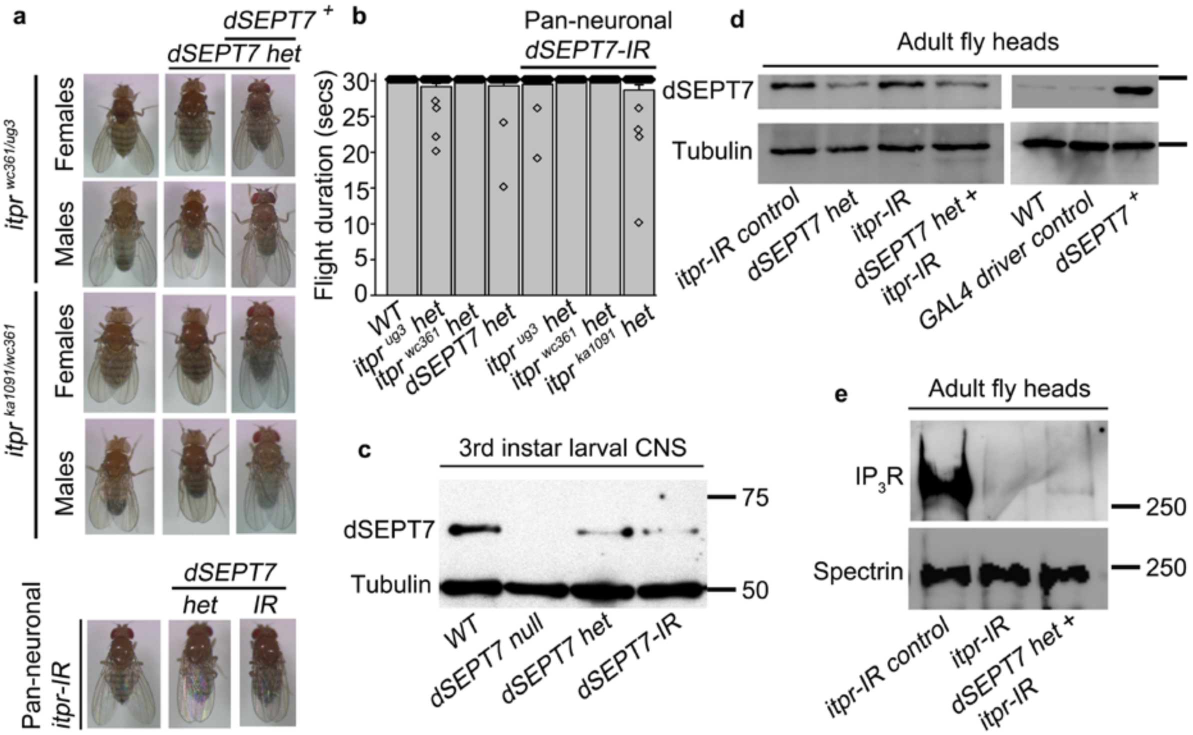

Supplementary Figure 1. **dSEPT7 reduction rescues wing posture in *itpr* mutant flies. (a)** Out-held wings observed in *itpr* mutants, *itpr*$^{wc361/ug3}$ and *itpr*$^{ka1091/wc361}$ (top), and in knockdown (*itpr-IR*, bottom) flies are rescued by dSEPT7 reduction. Over-expression of *dSEPT7*$^{+}$ leads to re-appearance of the wing posture defect in *itpr* mutant flies with reduced dSEPT7. **(b)** Flight durations in flies of the indicated genotypic controls. Each bar represents the mean flight time and each data point the flight duration for an individual fly (N=30). **(c, d)** dSEPT7 protein levels as detected in either the larval central nervous system (CNS) (c) or adult head extracts (d) of flies of the indicated genotypes .'GAL4 driver control' in (d) refers to flies with only the *elav*^{C155} *GAL4* and serves as a genetic control for the dSEPT7 overexpression using the pan-neuronal *elav*^{C155} *GAL4*. Tubulin is the loading control. **(e)** IP_3R protein level as detected in protein lysates from adult fly heads of the indicated genotypes. Spectrin was used as a loading control.

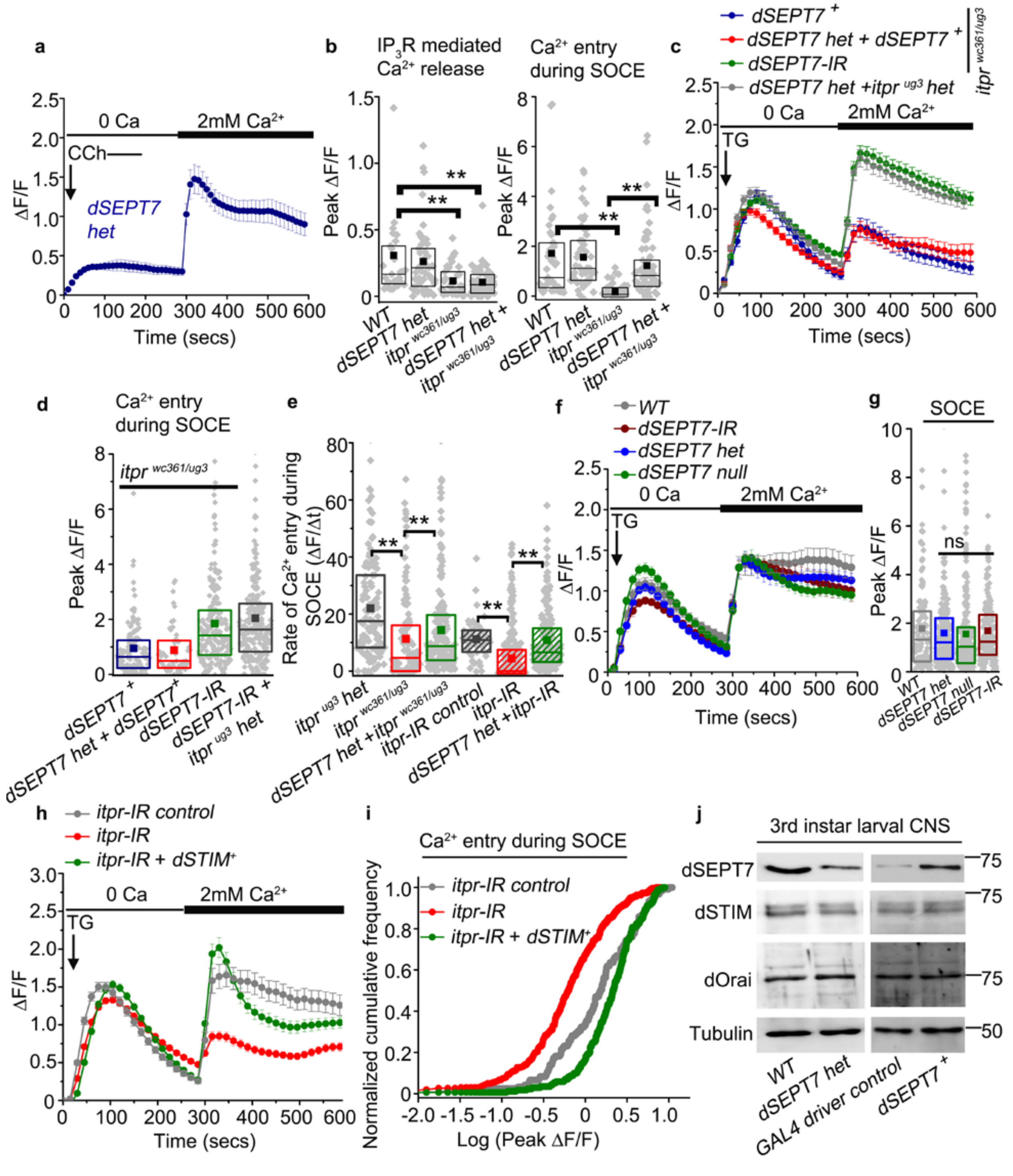

Supplementary Figure 2. dSEPT7 reduction does not affect SOCE and levels of dSTIM and dOrai (a) Changes in ΔF/F during Carbachol (CCh, 20μM) stimulated Ca^{2+} release and SOCE in neurons with dSEPT7 reduction. **(b)** Box plot quantifying peak

$\Delta F/F$ values during Ca^{2+} release through the IP_3R (left) and SOCE (right) in the indicated genotypes.**,$P<0.001$, Mann-Whitney U test with Bonferroni correction. **(c)** Changes in $\Delta F/F$ during TG (10μM) stimulated ER Ca^{2+} release and SOCE in neurons of the indicated genotypes. **(d)** Box plot quantifying peak $\Delta F/F$ values of SOCE in the indicated genotypes. **(e)** Quantification of the initial rate of Ca^{2+} entry during SOCE in the indicated genotypes. Initial rate of Ca^{2+} entry was calculated as $\Delta F/\Delta t$, where $\Delta F=F_{315}-F_{285}$ (computed from data shown in Fig. 2e and 2f) and Δt=30secs. **$P<0.001$, Mann-Whitney U test with Bonferroni correction. **(f)** Changes in $\Delta F/F$ during TG stimulated ER Ca^{2+} release and SOCE in neurons of the indicated genotypes. **(g)** Box plots quantifying peak values of $\Delta F/F$ during SOCE in the indicated genotypes. **(h)** Changes in $\Delta F/F$ during TG stimulated ER Ca^{2+} release and SOCE in neurons of the indicated genotypes. **(i)** K-S plots analyzing peak $\Delta F/F$ values during SOCE in the indicated genotypes. The distribution for cells with *itpr* knockdown is shifted to the left as compared to *itpr-IR* control, indicating higher proportion of cells with lower $\Delta F/F$ values; $P<0.001$, K-S test. Expression of *dSTIM*$^+$ shifts the distribution to the right, indicating higher proportion of cells with higher $\Delta F/F$ values; $P<0.0001$, K-S test. **(j)** Levels of dSEPT7, dSTIM and dOrai in lysates from the CNS of 3rd instar larvae of the indicated genotypes. Tubulin was the loading control.

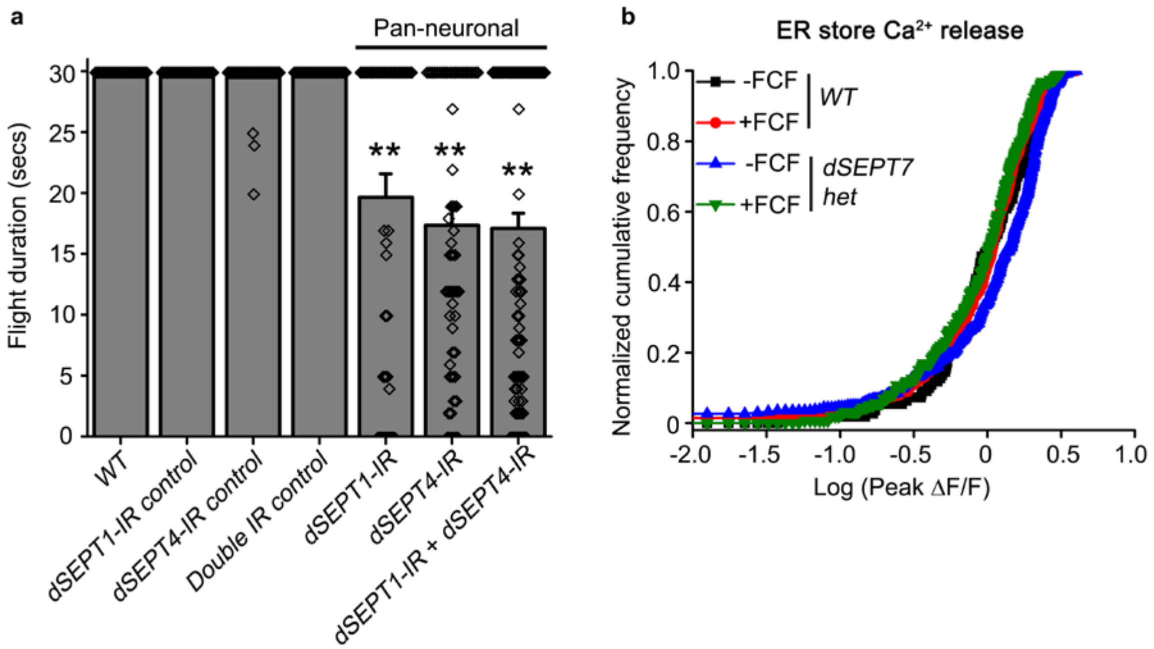

Supplementary Figure 3. dSEPT1 and dSEPT4 reduction in neurons results in flight deficits. (a) Flight duration in flies of the indicated genotypes. N $\geq$ 30. *Double-IR* control refers to animals with transgenic RNAi constructs for both genes but no GAL4. *elav*^{C155}*GAL4* was used for pan-neuronal knockdown of *dSEPT1* and *dSEPT4*. **P<0.01, Mann-Whitney U test with Bonferroni correction; Flight times of flies with pan-neuronal *dSEPT1-IR* or *dSEPT4-IR* compared to *dSEPT1-IR control* or *dSEPT4-IR control* respectively and double knockdown of *dSEPT1* and *dSEPT4* compared to the *Double-IR control*. **(b)** K-S plot analyzing the distribution of peak ΔF/F values obtained during TG-induced ER store release in the indicated genotypes and treatment conditions. All the distributions overlap indicating no significant differences between them.

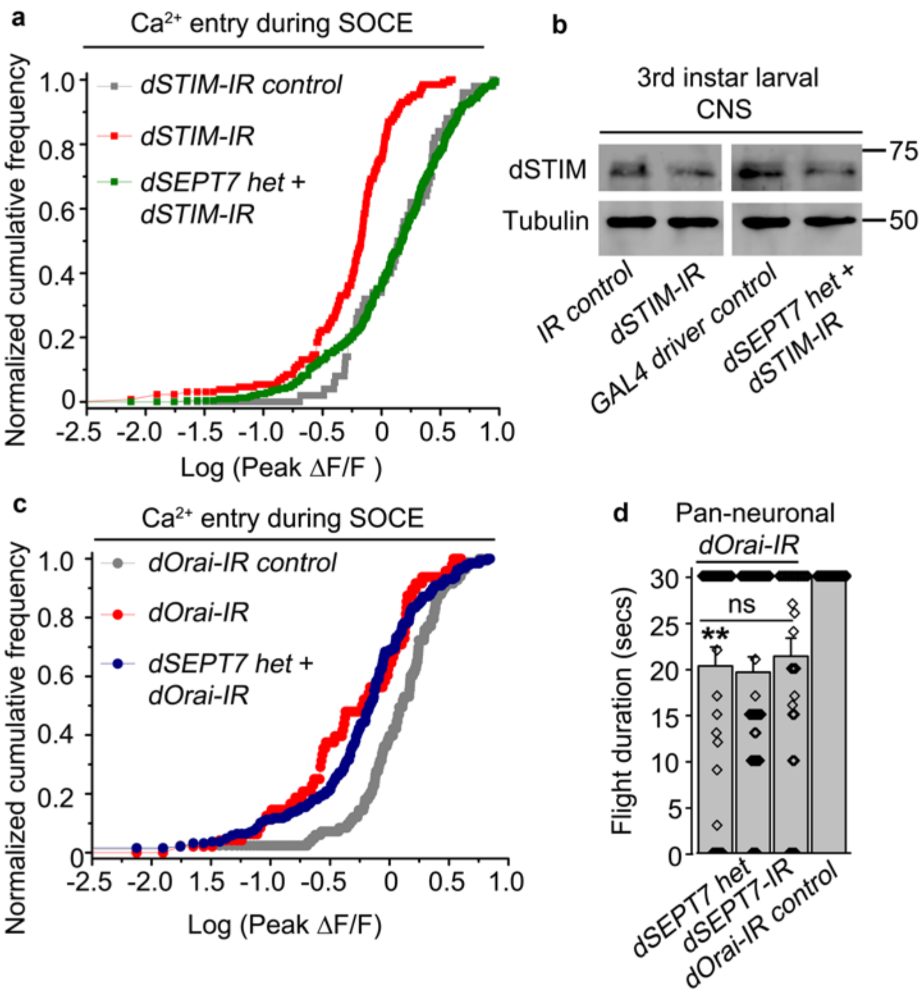

Supplementary Figure 4. dSEPT7 reduction fails to improve flight in flies with neuronal knockdown of dOrai. (a) K-S plot analyzing the distribution of peak ΔF/F values obtained during SOCE in the indicated genotypes. The distribution for *dSTIM-IR* is significantly shifted to the left compared to the *IR-control* indicating a higher proportion of cells with lower peak ΔF/F values in *dSTIM-IR*. P<0.001, K-S test. The distribution for *'dSEPT7 het+dSTIM-IR'* overlaps with the *IR-control* indicating no significant difference between the two. **(b)** Western blot quantifying the level of dSTIM protein in lysates prepared from the CNS of 3rd instar larvae of the indicated genotypes. Pan-neuronal *elav*^{C155}GAL4 was used to drive expression of the *dSTIM-IR*. *IR-control*

refers to the animals with the *dSTIM-IR* construct but no GAL4 resulting in no expression of the RNAi (*IR*). GAL4 driver control refers to animals with only the GAL4 driver and no *dSTIM-IR*. **(c)** K-S plot analyzing the distribution of peak ΔF/F values obtained during SOCE in the indicated genotypes. The distributions for *dOrai-IR* and *‘dSEPT7 het + dOrai-IR’* are significantly shifted to the left compared to the *IR-control* indicating higher proportion of cells with lower peak ΔF/F values in these genotypes. P<0.001, K-S test. **(d)** Mean flight durations in flies of the indicated genotypes. **P<0.001, Mann-Whitney U test, *dOrai-IR* compared to the *dOrai-IR control* and *‘dSEPT7 het + dOrai-IR’* and *‘dSEPT7-IR + dOrai-IR’* compared to *dOrai-IR*. N=30 flies.

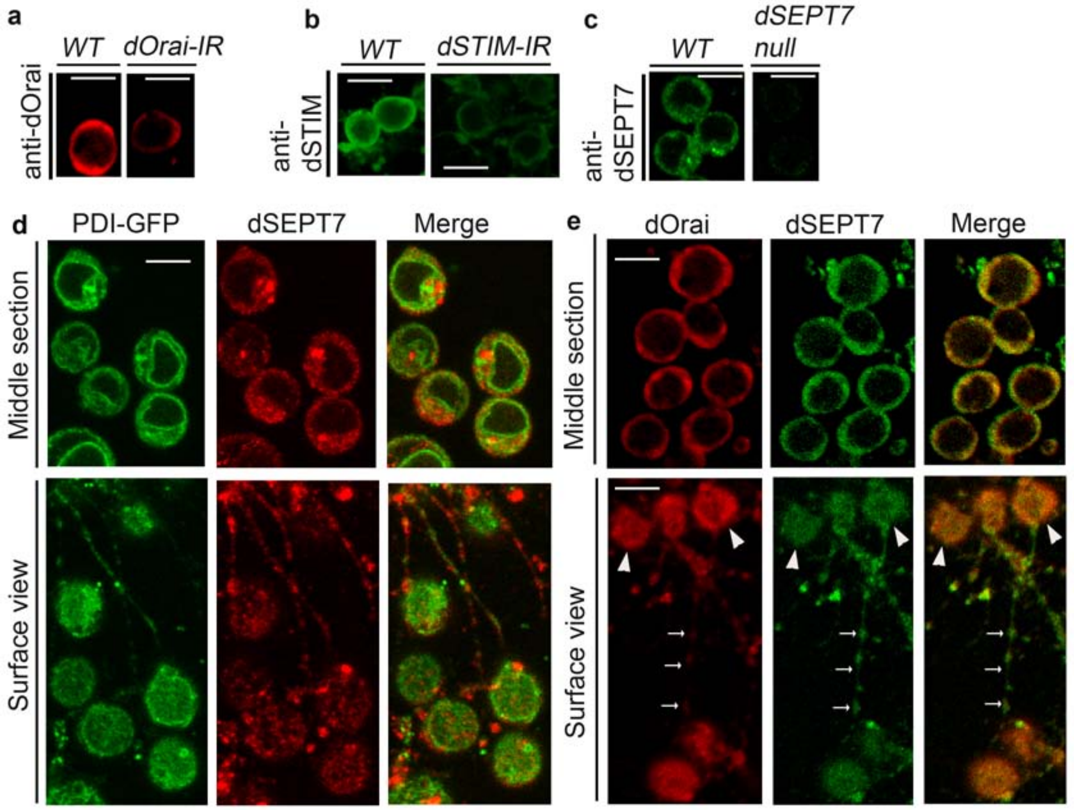

Supplementary Figure 5. Subcellular localization of dSEPT7 in primary *Drosophila* neurons. (a) dOrai**, (b)** dSTIM and **(c)** dSEPT7 immuno-reactivity in WT neurons and neurons with either *dOrai* knockdown (*dOrai-IR*) or *dSTIM* knockdown (*dSTIM-IR*) or *dSEPT7 null* neurons. **(d)** (Top panels) Confocal sections with localization of dSEPT7 (red) and the ER marker PDI-GFP (green) in the middle sections (~600 nm thick) of neuronal cell bodies. Pearson correlation coefficient (PCC) = 0.6903 $\pm$ 0.049, Mander's colocalization coefficient (MCC) for PDI-GFP in dSEPT7 = 0.70634 $\pm$ 0.081, MCC for dSEPT7 in PDI-GFP = 0.79107 $\pm$ 0.1032. Colocalization coefficients are represented as mean $\pm$ SEM. N> 50. (Bottom panels) Distribution of either dSEPT7 (red) or PDI-GFP (green, showing the cortical ER) on the cell surface by imaging an approximately 300nm thick optical section of neuronal cell bodies and neuronal projections. Pearson

correlation coefficient (PCC) = 0.56774 $\pm$ 0.051, Mander's colocalization coefficient (MCC) for PDI-GFP in dSEPT7 = 0.79234 $\pm$ 0.040, MCC for dSEPT7 in PDI-GFP = 0.9643 $\pm$ 0.01. **(e)** (Top panels) Confocal sections with localization of dOrai (red) and dSEPT7 (green) in the middle sections (~600 nm thick) of neuronal cell bodies. (Bottom panels) distribution of either dOrai (red) or dSEPT7 (green) on the cell surface (in an approximately 300nm thick optical section) of neuronal cell bodies (arrow heads) and neuronal projections (arrows). Regions of overlapping intensities appear yellow in the merged images**;** PCC = 0.85126 $\pm$ 0.0082; MCC for dSEPT7 in dOrai = 0.89376 $\pm$ 0.0233 and MCC for dOrai in dSEPT7 = 0.77652 $\pm$ 0.0427. Colocalization coefficients for dSEPT7/dOrai colocalization have been computed for the 'Surface view' only. Scale bar = 5μM. N$\geq$50 cells.

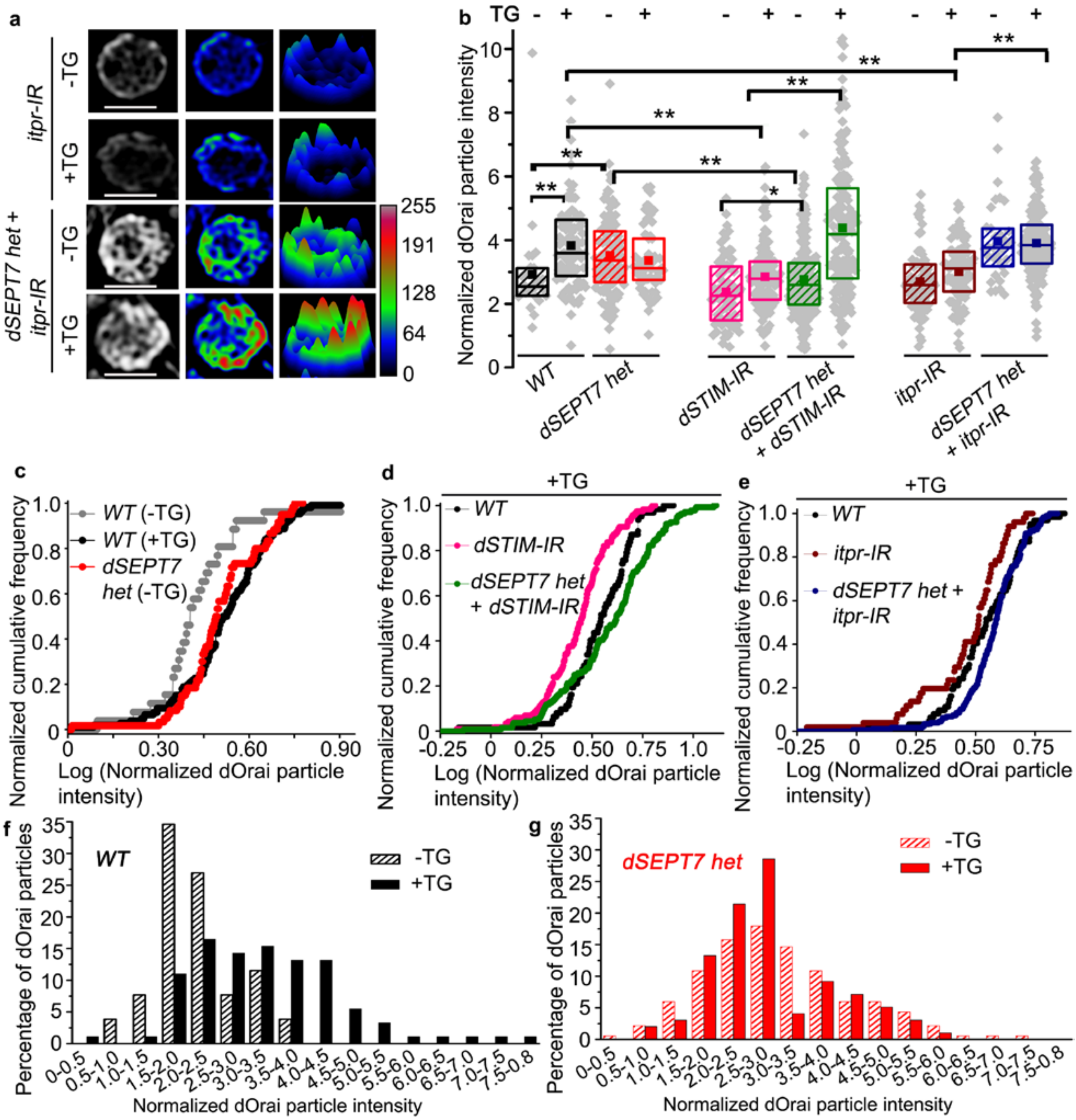

Supplementary Figure 6. Quantification of the dOrai clusters in neurons with reduced dSEPT7. (a) Representative images (after de-convolution) of dOrai distribution at the PM in resting or TG treated neurons of the indicated genotypes as original grey scale images (left panels) and pseudo-colored (middle panels). Warmer colors represent higher intensities. A surface plot constructed of the pseudo-colored image

shows the spatial distribution of dOrai intensities (right panels). **(b)** Box plot quantification of normalized dOrai intensities in the indicated genotypes and treatment conditions. Each data point represents the normalized dOrai intensity for a single dOrai particle. Normalized dOrai intensity=Intensity of dOrai particle/Total dOrai intensity at the PM; *P<0.01, **P<0.001, Mann-Whitney U test with Bonferroni correction; Brackets indicate the genotypes or treatment conditions being compared. **(c-e)** K-S plots analyzing the distribution of normalized intensities of dOrai particles in neurons of the indicated genotypes and treatment conditions. The distribution for neurons with reduced dSEPT7 **(c)** is significantly shifted to the right compared to the WT indicating a greater proportion of dOrai particles with higher normalized intensities**.** The distribution for *dSTIM-IR* **(d)** and *itpr-IR* **(e)** is significantly shifted to the left compared to WT indicating a higher proportion of dOrai particles with lower normalized intensities. P<0.001, K-S test for all the comparisons. **(f,g)** Bar graphs quantifying the proportion of dOrai particles of different normalized intensities observed in either resting (-TG) or TG treated (+TG) neurons of the indicated genotypes. The total range of normalized particle intensities has been binned into sizes of 0.5.

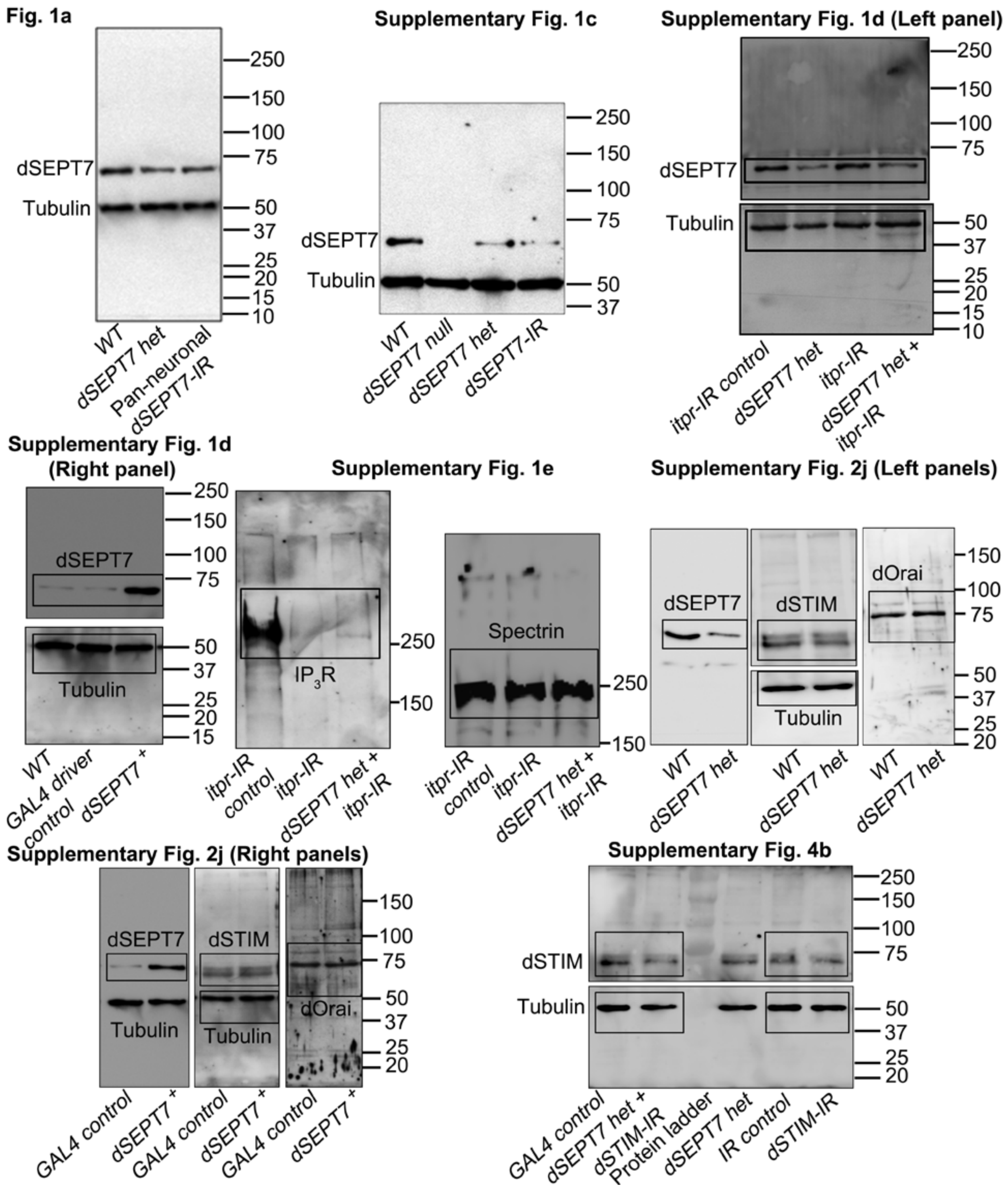

Supplementary Figure 7. Full size images of Western Blots. Boxes indicate the regions cropped for depiction in the Figures.
